# Quantitative proteome profiling reveals molecular hallmarks of egg quality in Atlantic halibut: impairments of transcription and protein folding impede protein and energy homeostasis during early development

**DOI:** 10.1186/s12864-022-08859-0

**Published:** 2022-09-07

**Authors:** Ozlem Yilmaz, Anders Mangor Jensen, Torstein Harboe, Margareth Møgster, Ragnfrid Mangor Jensen, Olav Mjaavatten, Even Birkeland, Endy Spriet, Linda Sandven, Tomasz Furmanek, Frode S. Berven, Anna Wargelius, Birgitta Norberg

**Affiliations:** 1 Institute of Marine Research, Austevoll Research Station, 5392 Storebø, Norway; 2 Department of Biomedicine, The Proteomics Facility of the University of Bergen (PROBE), 5009 Bergen, Norway; 3 Department of Biomedicine, The Molecular Imaging Center (MIC), University of Bergen, 5009 Bergen, Norway; 4 Institute of Marine Research, P.O. Box 1870, Nordnes, 5817 Bergen, Norway

**Keywords:** Egg quality, Atlantic halibut, Proteomics, Mitochondria, Mitochondrial DNA, Protein folding

## Abstract

**Background:**

Tandem mass tag spectrometry (TMT labeling-LC-MS/MS) was utilized to examine the global proteomes of Atlantic halibut eggs at the 1-cell-stage post fertilization. Comparisons were made between eggs judged to be of good quality (GQ) versus poor quality (BQ) as evidenced by their subsequent rates of survival for 12 days. Altered abundance of selected proteins in BQ eggs was confirmed by parallel reaction monitoring spectrometry (PRM-LC-MS/MS). Correspondence of protein levels to expression of related gene transcripts was examined via qPCR. Potential mitochondrial differences between GQ and BQ eggs were assessed by transmission electron microscopy (TEM) and measurements of mitochondrial DNA (mtDNA) levels.

**Results:**

A total of 115 proteins were found to be differentially abundant between GQ and BQ eggs. Frequency distributions of these proteins indicated higher protein folding activity in GQ eggs compared to higher transcription and protein degradation activities in BQ eggs. BQ eggs were also significantly enriched with proteins related to mitochondrial structure and biogenesis. Quantitative differences in abundance of several proteins with parallel differences in their transcript levels were confirmed in egg samples obtained over three consecutive reproductive seasons. The observed disparities in global proteome profiles suggest impairment of protein and energy homeostasis related to unfolded protein response and mitochondrial stress in BQ eggs. TEM revealed BQ eggs to contain significantly higher numbers of mitochondria, but differences in corresponding genomic mtDNA (*mt-nd5* and *mt-atp6*) levels were not significant. Mitochondria from BQ eggs were significantly smaller with a more irregular shape and a higher number of cristae than those from GQ eggs.

**Conclusion:**

The results of this study indicate that BQ Atlantic halibut eggs are impaired at both transcription and translation levels leading to endoplasmic reticulum and mitochondrial disorders. Observation of these irregularities over three consecutive reproductive seasons in BQ eggs from females of diverse background, age and reproductive experience indicates that they are a hallmark of poor egg quality. Additional research is needed to discover when in oogenesis and under what circumstances these defects may arise. The prevalence of this suite of markers in BQ eggs of diverse vertebrate species also begs investigation.

**Supplementary Information:**

The online version contains supplementary material available at 10.1186/s12864-022-08859-0.

## Background

Egg quality is of pivotal importance in biomedicine, agriculture, ecology and environmental science because of its tremendous influence on reproductive success or failure in all animals. Poor egg quality remains a serious problem attributed to various causes in human reproductive medicine [1, 2] and livestock production [3–5]. Maternal factors, primarily mRNA and proteins deposited in eggs during oogenesis are among the key influences on fertility and early embryogenesis. Recent research has increasingly focused on the motherlode of mRNA and proteins for clues to the origin of egg quality problems and their possible solutions [6–13]. The stockpile of maternal RNA and proteins drives early vertebrate development until activation of the zygotic genome around mid-blastula stage [14, 15]. Differential abundances of maternal transcripts may be indicators of quality in fish eggs [9], however, molecular changes involving modification of proteins after their uptake into growing oocytes also play crucial roles in many aspects of early development. These roles are not possible to elucidate using transcriptomic technologies alone. Applications of proteomics, an approach encompassing the dynamic transfer of genetic information into the actual effector molecules in the cell, are needed for elucidation of ongoing cellular events prior to zygotic genome activation. Despite the often limited consistency between transcript and product protein abundances [16], concurrent transcriptomic evaluations may help to reveal differences in steady-state cellular events that are related to egg quality during early stages of development.

Proteomic profiling has long been employed to study the cell biology of oocytes in many species, including humans, mice, pigs, fish and insects [17], as well as in investigations of egg quality in fishes such as rainbow trout (*Oncorhynchus mykiss*) [18], European sea bass (*Dicentrarchus labrax*) [19], Eurasian perch (*Perca fluviatilis*) [20], and hapuku (*Polyprion oxygeneios*) [21]. A comparison of the global proteomes of different quality eggs from zebrafish revealed a number of proteins as potential quality markers and, more importantly, several molecular mechanisms and related physiological processes associated with egg quality in this species [22]. Furthermore, a recent study revealed consecutive changes in the global proteome of 1-cell-stage zebrafish eggs after knock out (KO) of several genes encoding vitellogenins (*vtg1, 4* and *5*; *vtg1*-KO and *vtg3*; *vtg3*-KO) using CRISPR/Cas9 genome editing technology [23, 24]. Taken together, the results delivered a clear portrait of impaired molecular mechanisms that impacted egg quality in zebrafish, as judged by offspring developmental competence, with striking similarities between *vtg*-KO and poor quality egg proteome profiles.

Despite species-specific differences in physiological aspects of early development, the evolutionarily conservation of cellular events led us to investigate whether the prior findings in zebrafish are applicable to egg quality in marine species of aquaculture interest. The Atlantic halibut (*Hippoglossus hippoglossus*) is highly prized in global fish markets, with decreasing landings in capture fisheries and increasing demand for farm production is considered as a representative of such species. Notwithstanding the progress in research and cultivation efforts that has been made, several bottlenecks persist, including an unsteady supply of high quality eggs and fry. In southwestern Norway, vitellogenesis in halibut females commences in August/September, and proceeds through spawning, which occurs between February and April [25]. A well-composed broodstock diet is important to ensure that nutritional requirements for gametogenesis are met so as to optimize gamete quality. In addition, as female breeders are generally kept for utilization over several consecutive spawning seasons, proper feeding routines are crucial to support ovarian recrudescence, oogenesis and vitellogenesis occurring over a number of years.

Halibut females in captivity can release 6–12 batches of eggs, at 2–3 days intervals, with a fecundity of 13,000–15,000 eggs ^.^kg^− 1^, or up to 1 million eggs per female [26]. However, eggs are often of highly variable quality. Taken together, these features make Atlantic halibut an attractive candidate for studying molecular determinants of egg quality.

The objectives of this study were 1) to reveal the proteomic profiles of good versus poor quality Atlantic halibut eggs, 2) to identify proteins that can serve as egg quality markers, and 3) to discover molecular mechanisms determining egg quality using powerful next generation proteomic approaches including tandem mass tag (TMT) labeling- and parallel reaction monitoring (PRM)-based liquid chromatography tandem mass spectrometry (LC-MS/MS). Discovery of such mechanisms in poor quality eggs will spur development of practical strategies to identify and eliminate the potential causes of egg quality problems in Atlantic halibut and other farmed fish species, thereby contributing to development of effective strategies for improving breeding practices and sustainable growth of Norwegian and global aquaculture. These developments will also contribute to advances in reproductive biology of other organisms, such as livestock and humans, that share many common properties of reproduction and early development.

## Results

### Assessment of reproductive parameters

Based on our overall experience in hatchery practices, the cumulative percentage of surviving embryos stabilizes prior to hatching, by 12 days post fertilization (dpf). Therefore, embryo survival at this stage was utilized as the measure of egg quality in this study. The actual survival rates in the overall sample inventory ranged from 93% for good quality eggs to 25% for the poor quality eggs. Egg batches with an embryo survival rate of ≥72% were considered to be of good quality and those spawns with ≤71% embryo survival were considered to be of poor quality in all seasons. Egg batches from halibut females employed in this study showed high variation in fecundity, buoyancy, fertilization, and normal cell division. Some egg batches exhibited low embryo survival rates despite a high percentage of fertilization and embryo progression through early stages of cell division (see batches marked with asterisks in Table S1). Correlation assessments between other measured reproductive parameters (female fecundity, egg buoyancy, fertilization rate, normal cell division) and embryo survival rate at 12 dpf were made using Spearman’s correlation analysis. Results indicate no obvious link of fecundity and egg buoyancy but strong correlation of fertilization rate and normal cell division rate to the proportion of embryos surviving before hatching up to 12 dpf (*p* < 0.01, Fig. S1, Panel A). When the same parameters were subjected to Spearman’s correlation with egg quality ascertained in this way, the stated relationships were apparent and the range of observations for each parameter in the two egg quality groups were made readily evident (*p* < 0.01, Fig. S1, Panel B). The range of survival observations for good and poor quality eggs, and a summary of the referenced Spearman’s correlation coefficients and corresponding significance values are shown in Fig. S1, Panel C.

### TMT labeling based LC-MS/MS

**Fig. 1:**
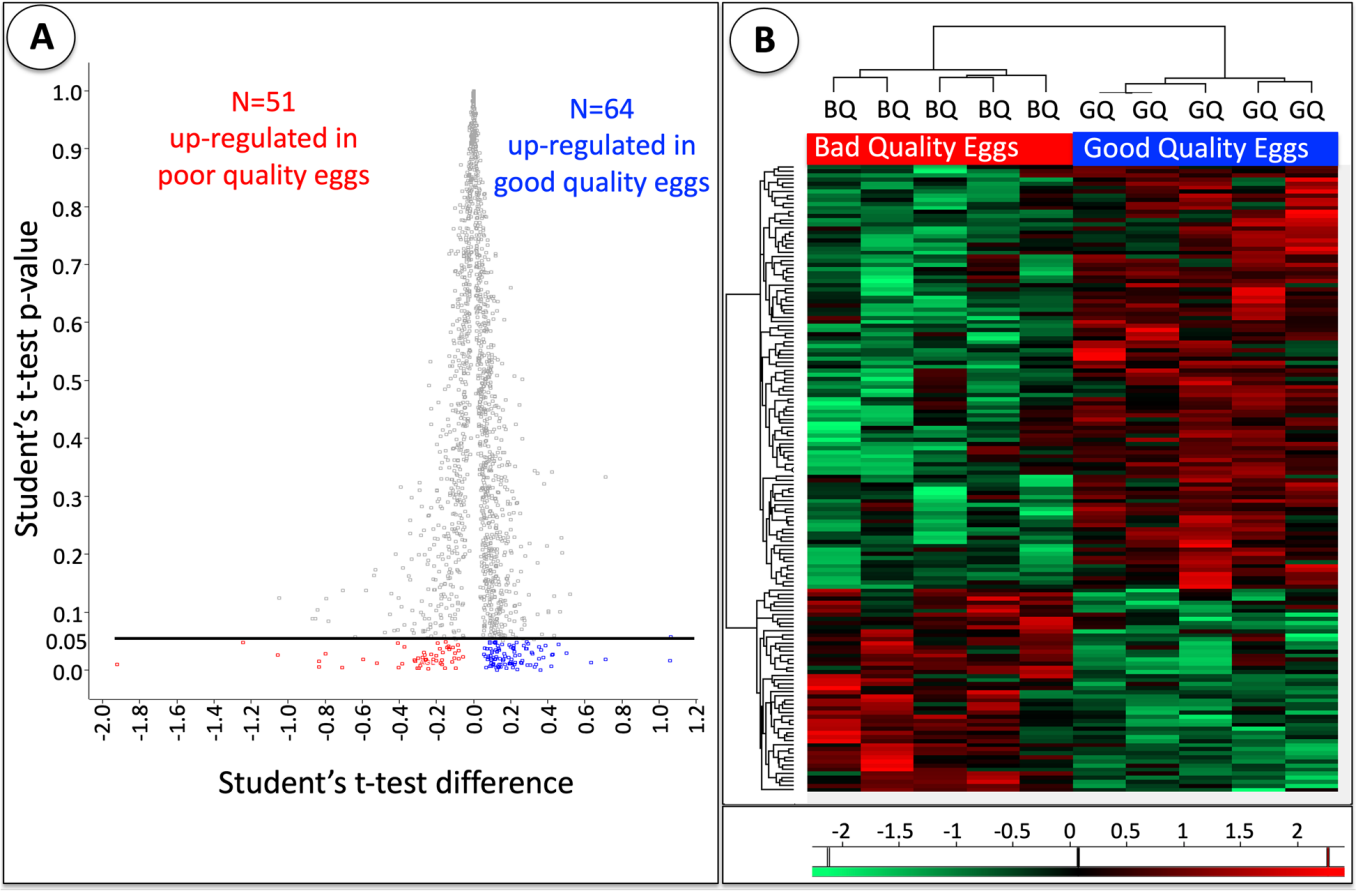
Proteins differentially abundant between good and poor quality halibut eggs. Panel **A** Representation of differential abundance for 115 proteins detected by TMT labeling based LC-MS/MS based on the significance of differences assessed by Student’s t-tests. Y axis indicates *p* values while X axis represents test differences. Proteins up-regulated in poor quality eggs (*N* = 51) are indicated in red while those up-regulated in good quality eggs (therefore down-regulated in poor quality eggs, *N* = 64) are indicated in blue. The black horizontal line above red and blue markers represent the separation of differentially abundant proteins retained after the *p* < 0.05 cut off value. A complete list of these proteins along with detailed information on their NCBI gene IDs, NCBI accession numbers, associated protein names from the human database, protein full names, functional categories (according to Fig. 2), significance of differences in abundance (Independent t- test *p* < 0.05 followed by Benjamini Hochberg correction for multiple tests *p* < 0.05), relative abundance ratios (GQ/BQ and BQ/GQ, respectively), and regulation tendencies (BQ-upregulated or BQ-downregulated) are given in Table S2. Panel **B.** A heatmap clustering of differentially abundant proteins between good and poor quality egg groups

A total of 1619 out of 1886 identified proteins were considered to be valid if they were detected in at least four biological samples. A total of 115 valid proteins were found to be differentially abundant between good and poor quality eggs (Independent samples t-test, *p* < 0.05 followed by Benjamini Hochberg correction for multiple testing, *p* < 0.05). Detailed information on these proteins is given in Table S2. In this study, proteins with higher abundance in good quality eggs are indicated as down-regulated in poor quality eggs (*N* = 64), and those with higher abundance in poor quality eggs are indicated as up-regulated in poor quality eggs (*N* = 51). Fig. 1A shows a volcano plot of these proteins based on *p* values obtained from Student’s t-test, *p* < 0.05 followed by Benjamini Hochberg correction for multiple testing, *p* < 0.05. A heatmap representation of the clustering of differentially regulated proteins based on their abundance in good versus poor quality eggs is given in Fig. 1B.

**Fig. 2:**
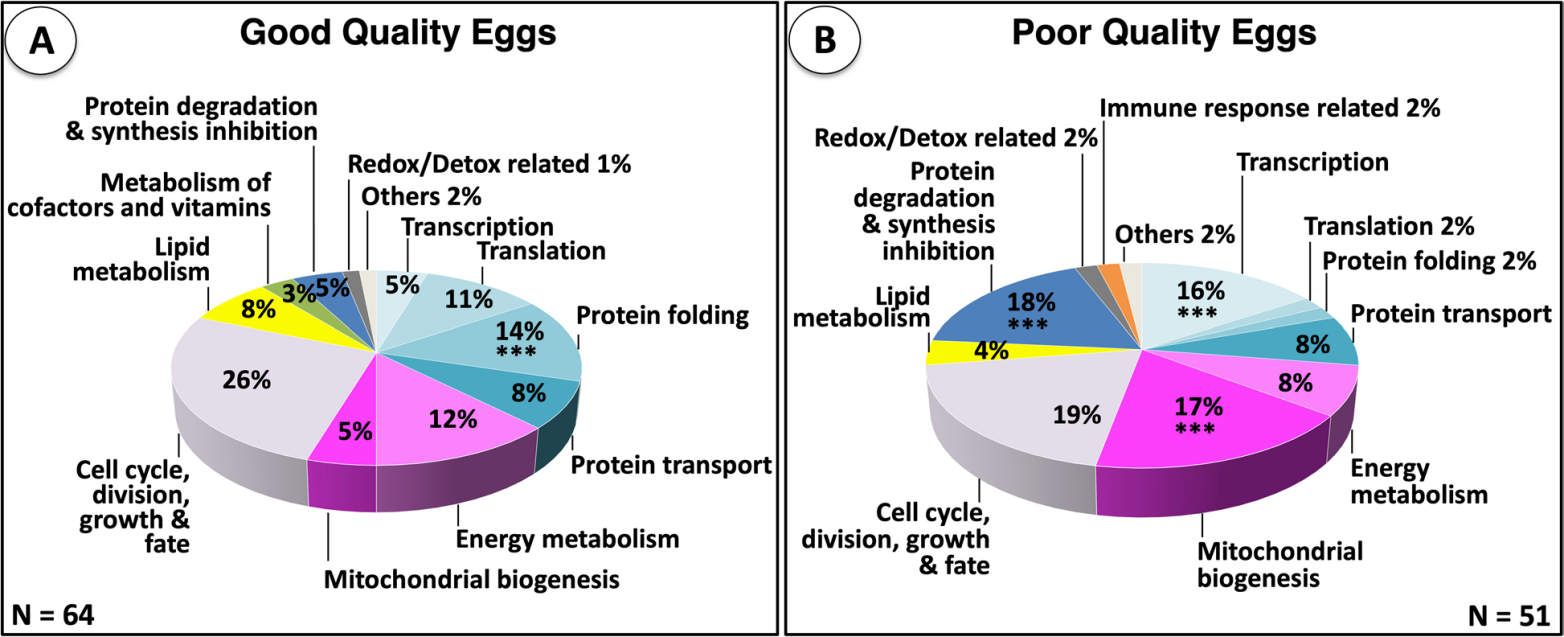
Distribution of differentially abundant proteins among functional categories. Panel **A** Proteins up-regulated in good quality eggs (*N* = 64) and, therefore down-regulated in poor quality eggs. Panel **B.** Proteins up-regulated in poor quality eggs (*N* = 51). The overall distribution of differentially regulated proteins among functional categories significantly differed between good and poor quality eggs (χ^2^, *p* < 0.05). Asterisks indicate significant differences between different groups in the proportion of differentially regulated proteins within a functional category (χ^2^, *p* < 0.05). The corresponding NCBI gene IDs, NCBI accession numbers, associated protein names from the human database, protein full names, functional categories (shown above), significance of differences in abundance (Independent t- test *p* < 0.05 followed by Benjamini Hochberg correction for multiple tests *p* < 0.05), relative abundance ratios (GQ/BQ and BQ/GQ, respectively), and regulation tendencies (BQ-upregulated or BQ-downregulated) are given in Table S2

Frequency distributions of differentially abundant proteins among thirteen arbitrarily chosen functional categories accounting for > 90% of the proteins are shown in Fig. 2. Proteins that were down-regulated in poor quality eggs (*N* = 64) (Fig. 2 Panel A) were mainly related to cell cycle, division, growth and fate (26%), protein folding (14%), energy metabolism (12%), translation (11%), protein transport (8%), and lipid metabolism (8%) with the remaining categorized proteins being related to protein degradation and synthesis inhibition (5%), transcription (5%), mitochondrial biogenesis (5%), metabolism of cofactors and vitamins (3%), and redox/detox activities (1%). Only 2 % of proteins that were down-regulated in poor quality eggs were placed in the category ‘others’. Proteins that were up-regulated in poor quality eggs (*N* = 51) (Fig. 2 Panel B) were mainly related to cell cycle, division, growth and fate (19%), protein degradation and synthesis inhibition (18%), mitochondrial biogenesis (17%), transcription (16%), energy metabolism (8%), and protein transport (8%) with the remaining categorized proteins being related to lipid metabolism (4%), protein folding (2%), translation (2%), redox/detox activities [2], and immune response related (2%). Two percent of proteins that were up-regulated in poor quality eggs were placed in the category ‘others’. The distribution of these differentially regulated proteins among functional categories significantly differed between egg quality groups (χ^2^ *p* < 0.05). Accordingly, good quality eggs seem to contain a significantly higher proportion of proteins related to protein folding (14%), while poor quality eggs contain significantly higher proportions of proteins related to transcription (16%), protein degradation and synthesis inhibition (18%), and mitochondrial biogenesis (17%) (Fig. 2).

**Fig. 3:**
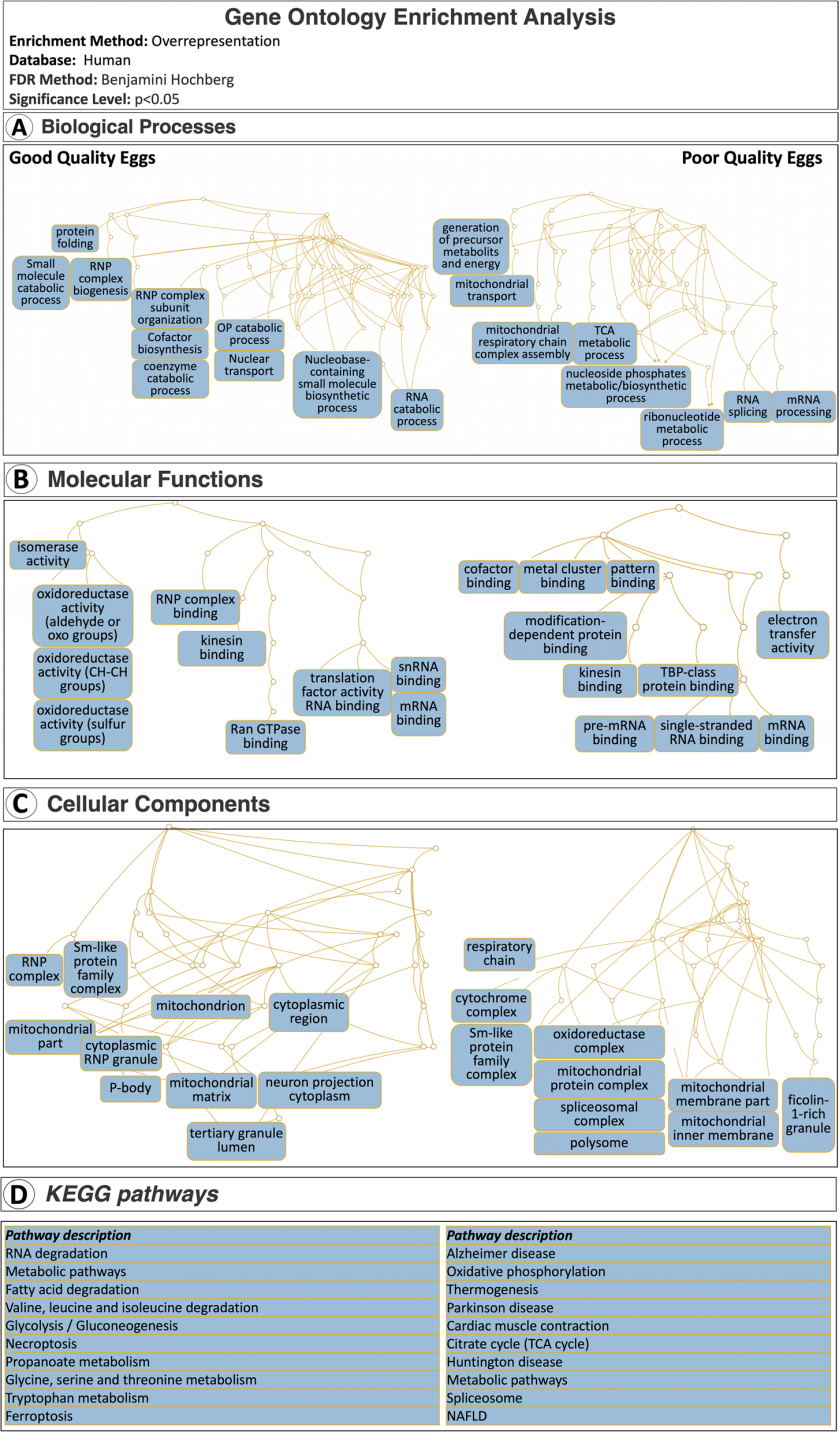
Gene ontology overrepresentation-based enrichment analyses for differentially abundant proteins. Panel **A.** Biological processes significantly enriched in good quality eggs (Left) versus in poor quality eggs (Right). Panel **B**. Molecular functions significantly enriched in good quality eggs (Left) versus in poor quality eggs (Right). Panel **C**. Cellular components significantly enriched in good quality eggs (Left) versus in poor quality eggs (Right). Panel **D**. KEGG pathways significantly enriched in good quality eggs (Left) versus in poor quality eggs (Right). A total of *N* = 51 and *N* = 64 proteins up- or down-regulated in poor quality eggs, respectively, were mapped against the human database for enrichment analyses using the overrepresentation method at *p* < 0.05 followed by Benjamini Hochberg correction for multiple tests (*p* < 0.05). Images used in this figure were obtained from WebGestalt, a free functional enrichment analysis web tool and they are not subject to copyright

Gene ontology (GO) enrichment analyses based on overrepresentation tests (*p* < 0.05), with the human database used as a reference, revealed significant Biological processes, Molecular functions and Cellular components with a close relation to findings of the frequency distribution analyses (Fig. 3). Biological processes that were overrepresented by proteins down-regulated in poor quality eggs were as follows; protein folding, small molecule catabolic process, ribonucleoprotein (RNP) complex biogenesis, RNP complex subunit organization, cofactor biosynthetic process, coenzyme metabolic process, organophosphate (OP) catabolic process, nuclear transport, nucleobase-containing small molecule biosynthetic process, and RNA catabolic process. Molecular functions overrepresented by proteins down-regulated in poor quality eggs were related to isomerase activity, oxidoreductase activity (acting on the aldehyde or oxo group of donors), oxidoreductase activity (acting on the CH-CH group of donors), oxidoreductase activity (acting on a sulfur group of donors), RNP complex binding, kinesin binding, translation factor activity (RNA binding), snRNA binding, mRNA binding, and Ran GTPase binding. Cellular components overrepresented by these proteins were RNP complex, Sm-like protein family complex, mitochondrion, cytoplasmic region, mitochondrial part, cytoplasmic RNP granule, P-body, mitochondrial matrix, neuron projection cytoplasm, and tertiary granule lumen. KEGG pathways that were significantly overrepresented by this same set of proteins were RNA degradation, metabolic pathways, fatty acid degradation, valine, leucine and isoleucine degradation, glycolysis/gluconeogenesis, necroptosis, propanoate metabolism, glycine, serine and threonine metabolism, tryptophane metabolism, and ferroptosis (Fig. 3A, B, C and D Left sides).

In contrast, Biological processes that were overrepresented by proteins up-regulated in poor quality eggs were as follows; generation of precursor metabolites and energy, mitochondrial transport, mitochondrial respiratory chain complex assembly, tricarboxylic acid metabolic process, nucleoside phosphates metabolic/biosynthetic process, ribonucleotide metabolic process, RNA splicing, and mRNA processing. Molecular functions overrepresented by proteins up-regulated in poor quality eggs were related to cofactor binding, metal cluster binding, pattern binding, modification-dependent protein binding, kinesin binding, TBP-class protein binding, electron transfer activity, pre-mRNA binding, single-stranded RNA binding, and mRNA binding. Cellular components overrepresented by these proteins were respiratory chain, cytochrome complex, Sm-like protein family complex, oxidoreductase complex, mitochondrial protein complex, spliceosomal complex, polysome, mitochondrial membrane part, mitochondrial inner membrane and ficolin-1-rich granule. KEGG pathways that were significantly overrepresented by the same set of proteins were Alzheimer’s disease, oxidative phosphorylation, thermogenesis, Parkinson’s disease, cardiac muscle contraction, citrate cycle (TCA cycle), Huntington disease, metabolic pathways, spliceosome, and non-alcoholic fatty liver disease (NAFLD) (Fig. 3A, B, C and D Right sides). Taken together, the congruent results of the GO enrichment analyses for Biological processes, Molecular functions, Cellular components and KEGG pathways clearly indicate for a struggle of the BQ eggs in RNA processing along with mitochondria generation and functioning.

**Fig. 4:**
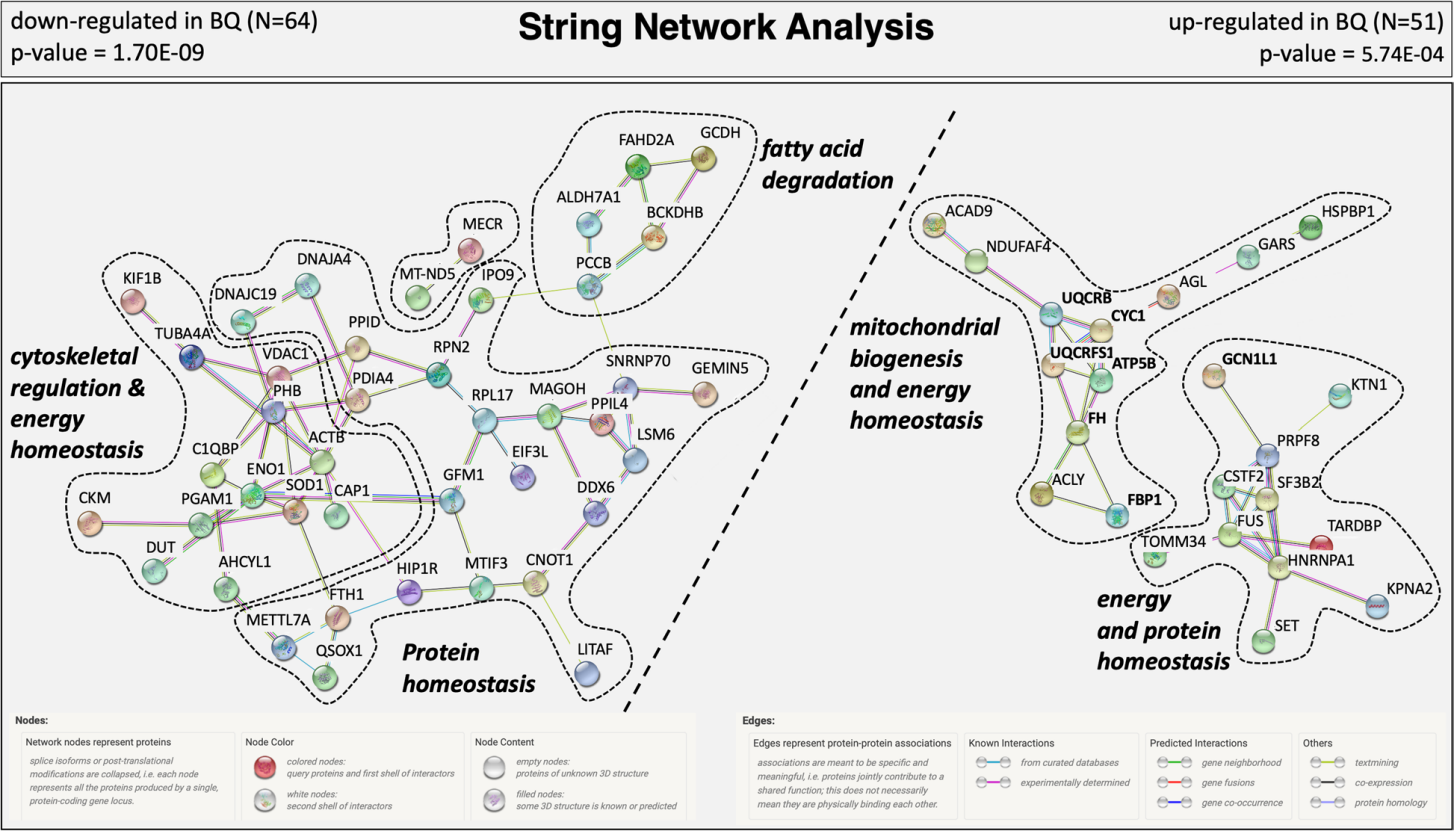
Protein-protein interaction network analysis of the differentially abundant proteins. Network clusters are given for *N* = 64 proteins which were down-regulated in poor quality (BQ) eggs and *N* = 51 proteins which were up-regulated in BQ eggs. The subnetworks formed by proteins down-regulated in BQ eggs are shown to the left of the bold diagonal dashed line, and the subnetworks formed by proteins up-regulated in BQ eggs are shown to the right of this line. Where possible, finely dashed lines encircle clusters of interacting proteins involved in physiological processes distinct from other such clusters. Network nodes (spheres) represent all proteins originated from a single, protein-coding gene locus excluding splice isoforms or post-translational modifications, and each are named for the human proteins to which spectra were mapped (see Table S2 for full protein names). Specific and meaningful protein-protein associations are indicated by edges (colored lines). Model statistics are presented at the top left and at the top right of the figure for proteins down- and up-regulated in BQ eggs, respectively. Images used in this figure were obtained from STRING a free biological database and web source for predicted protein–protein interactions analyses and they are not subject to copyright

When the 115 differentially regulated proteins with significant differences in abundance between good and poor quality eggs were submitted separately (down-regulated in BQ, *N* = 64, up-regulated in BQ, *N* = 51) to a functional protein association network analysis using the Search Tool for the Retrieval of Interacting Genes/Proteins (STRING) and the human protein database, they resolved into a network with significantly and substantially greater numbers of known and predicted interactions between proteins than would be expected of the same size lists of proteins randomly chosen from the human database (Fig. 4). The subnetwork formed by proteins down-regulated in poor quality eggs is made up of three interrelated clusters mainly related to cytoskeletal regulation and energy and protein homeostasis (Fig. 4 Left side). The cluster to the far left includes proteins involved in cytoskeletal organization such as Adenylyl cyclase-associated protein 1 (CAP1), Actin beta (ACTB), Tubulin alpha 4a (TUBA4A), Kinesin family member 1B (KIF1B), Voltage dependent anion channel 1 (VDAC1), Deoxyuridine 5′-triphosphate nucleotidohydrolase, mitochondrial (DUT), and Adenosylhomocysteinase like 1 (AHCYL1), and in energy production and homeostasis such as Creatine kinase (M-type) (CKM), Phosphoglycerate mutase 1 (PGAM1), and Enolase (ENO1). Other proteins forming this cluster are the Complement component 1 Q subcomponent-binding protein, mitochondrial (C1QBP) and Prohibitin (PHB), which are related to mitochondrial structure, and Superoxide dismutase 1 (SOD1), which is related to redox/detox activities.

The central cluster in this subnetwork includes proteins related to mRNA biogenesis and transcription (LSM6 homolog, U6 small nuclear RNA and mRNA degradation associated (LSM6), ATP-dependent RNA helicase DDX6 (DDX6), Small nuclear ribonucleoprotein U1 subunit 70 (SNRNP70), and Mago homolog, exon junction complex subunit (MAGOH)), protein translation (Gem-associated protein 5 (GEMIN5), Eukaryotic translation initiation factor 3 subunit L (EIF3L), Ribosomal protein L17 (RPL17), G elongation factor mitochondrial 1 (GFM1), and Translation initiation factor IF-3, mitochondrial (MTIF3)), protein folding (Dolichyl-diphosphooligosaccharide-protein glycosyltransferase subunit 2 (RPN2), Methyltransferase like 7A (METTL7A), Peptidylprolyl isomerase like 4 (PPIL4), DnaJ heat shock protein family (Hsp40) member A4 (DNAJA4), DnaJ heat shock protein family (Hsp40) member C19 (DNAJC19), Peptidylprolyl isomerase D (PPID), Protein disulfide isomerase family A member 4 (PDIA4), Quiescin sulfhydryl oxidase 1 (QSOX1)), protein transport (Importin (IPO9)) and Huntingtin-interacting protein 1-related protein (HIP1R)). Three other proteins included in this cluster are CCR4-NOT transcription complex subunit 1 (CNOT1), a transcription suppressor associated with DNA damage, Lipopolysaccharide-induced tumor necrosis factor-alpha factor homolog (LITAF), which targets proteins for lysosomal degradation, and Ferritin heavy chain 1 (FTH1), which is related to cellular iron homeostasis. The cluster in the upper right of this subnetwork includes proteins whose major functions are mostly related to fatty acid degradation (Propionyl-CoA carboxylase beta chain, mitochondrial (PCCB), Alpha-aminoadipic semialdehyde dehydrogenase (ALDH7A1), Glutaryl-CoA dehydrogenase, and mitochondrial (GCDH)), and amino acid catabolism in mitochondria (2-oxoisovalerate dehydrogenase subunit beta, mitochondrial (BCKDHB)), and a redox factor employed for respiration in the electron transport chain (Fumarylacetoacetate hydrolase domain containing 2A (FAHD2A)).

Proteins that were found to be up-regulated in poor quality eggs formed a subnetwork made up of two major clusters (Fig. 4 Right side). The cluster on the top left includes proteins mainly involved in mitochondrial structure (Ubiquinol-cytochrome c reductase binding protein (UQCRB), Cytochrome b-c1 complex subunit Rieske, mitochondrial (UQCRFS1), Cytochrome c1 (CYC1), and ATP synthase F1 subunit beta (ATPF5B)), complex assembly factors (Complex I assembly factor ACAD9, mitochondrial (ACAD9), NADH:ubiquinone oxidoreductase complex assembly factor 4 (NDUFAF4)), and proteins related to mitochondrial energy generation (Fumarate hydratase, mitochondrial (FH), ATP citrate lyase (ACLY), Fructose-bisphosphatase 1 (FBP1), and Glycogen debranching enzyme (AGL)). This cluster includes two other proteins, Glycine-tRNA ligase (GARS), which is related to protein translation, and Hsp70-binding protein 1 (HSPBP1), which is related to protein degradation and synthesis inhibition. The second cluster shown in the bottom right of this subnetwork includes proteins mainly related to mRNA biogenesis and transcription (Cleavage stimulation factor subunit 2 (CSTF2), Splicing factor 3b subunit 2 (SF3B2), RNA-binding protein FUS (FUS), Pre-mRNA processing factor 8 (PRPF8), Heterogeneous nuclear ribonucleoprotein A1 (HNRNPA1) and TAR DNA binding protein (TARDBP)). Some other proteins within this cluster are the Mitochondrial import receptor subunit TOM34 (TOMM34) and Kinectin 1 (KTN1), which are involved in mitochondrial biogenesis, Karyopherin subunit alpha 2 (KPNA2), a nuclear protein importer, the GCN1 activator of EIF2AK4 (GCN1) related to protein degradation and synthesis inhibition, and the SET nuclear proto-oncogene (SET) involved in DNA replication and chromatin binding.

Enrichment results for the revealed networks are given in Table S3. Aside from being in complete accordance with findings of the GO enrichment analyses for Biological processes, Molecular functions and Cellular components, these results include some interesting KEGG and Reactome pathway enrichment signatures. On the one hand, proteins down-regulated in poor quality eggs were enriched in metabolic pathways, RNA degradation, valine, leucine, and isoleucine degradation, fatty acid degradation, necroptosis and glycolysis/gluconeogenesis KEGG pathways, and also the metabolism Reactome pathway (PPI network enrichment value *p* = 1.70 × 10^− 9^). On the other hand, proteins up-regulated in poor quality eggs were enriched in Alzheimer’s disease, Parkinson’s disease, thermogenesis, metabolic pathways, oxidative phosphorylation, cardiac muscle contraction, Huntington’s disease, citrate cycle (TCA cycle), spliceosome and non-alcoholic fatty liver disease (NAFLD) KEGG pathways, and also the citric acid (TCA) cycle and respiratory electron transport, ATP synthesis by chemiosmotic coupling, and heat production by uncoupling proteins, respiratory electron transport, processing of capped intron containing pre-mRNA, mRNA splicing - major pathway, metabolism, ISG 15 antiviral mechanism, and metabolism of RNA Reactome pathways (PPI network enrichment value *p* = 0.000574).

Taking into account the overall results obtained by TMT labeling-based LC-MS/MS, a total of 21 proteins that significantly differed in abundance between good and poor quality eggs were chosen as candidate markers of egg quality in this study. Thirteen proteins down-regulated in poor quality eggs were chosen to represent the majority of functional categories, with a special emphasis on proteins related to mitochondrial biogenesis and energy metabolism related proteins. The remaining proteins up-regulated in poor quality eggs were chosen to mainly represent the functional categories of mitochondrial biogenesis and energy metabolism. Fold differences in abundance of candidate proteins between good and poor quality eggs varied between 1.07 and 1.85 for proteins down-regulated in poor quality eggs and between 1.07 and 4.67 for proteins up-regulated in poor quality eggs. Comparisons between good and poor quality halibut eggs in the abundance of these proteins are given in Fig. S2.

### qPCR

Gene expression levels for the 20 candidate markers with a significant difference in protein abundance between good and poor quality eggs, in addition to *mt-atp6*, a mitochondrial gene utilized as marker for high quality eggs in previous studies [13], are given in Fig. S3. Eight out of the 21 genes (*cyc1*, *fh*, *uqcrb*, *gcn1*, *ghitm*, *uqcrfs1*, *fbp1a*, and *atp5f1a*) exhibited increases in expression in poor quality eggs coinciding with increased abundance of the product protein and with significant differences between good and poor quality eggs (Independent samples t-test, *p* < 0.05 followed by Benjamini Hochberg correction for multiple testing, *p* < 0.05). Four genes (*mt-nd5*, *mt-atp6*, *acly1*, and *dhrs9*) appeared to show increased expression in poor quality eggs, the same tendency shown by product protein abundance, but these differences were not statistically significant. Finally, nine genes (*gcdh*, *ppid*, *gatd3a*, *gfm1*, *cap1*, *phb*, *sod1*, *mecr*, and *vdac*) appeared to exhibit changes in expression converse to the trend of product protein abundance, and these differences were also not statistically significant.

### PRM based LC-MS/MS

**Fig. 5:**
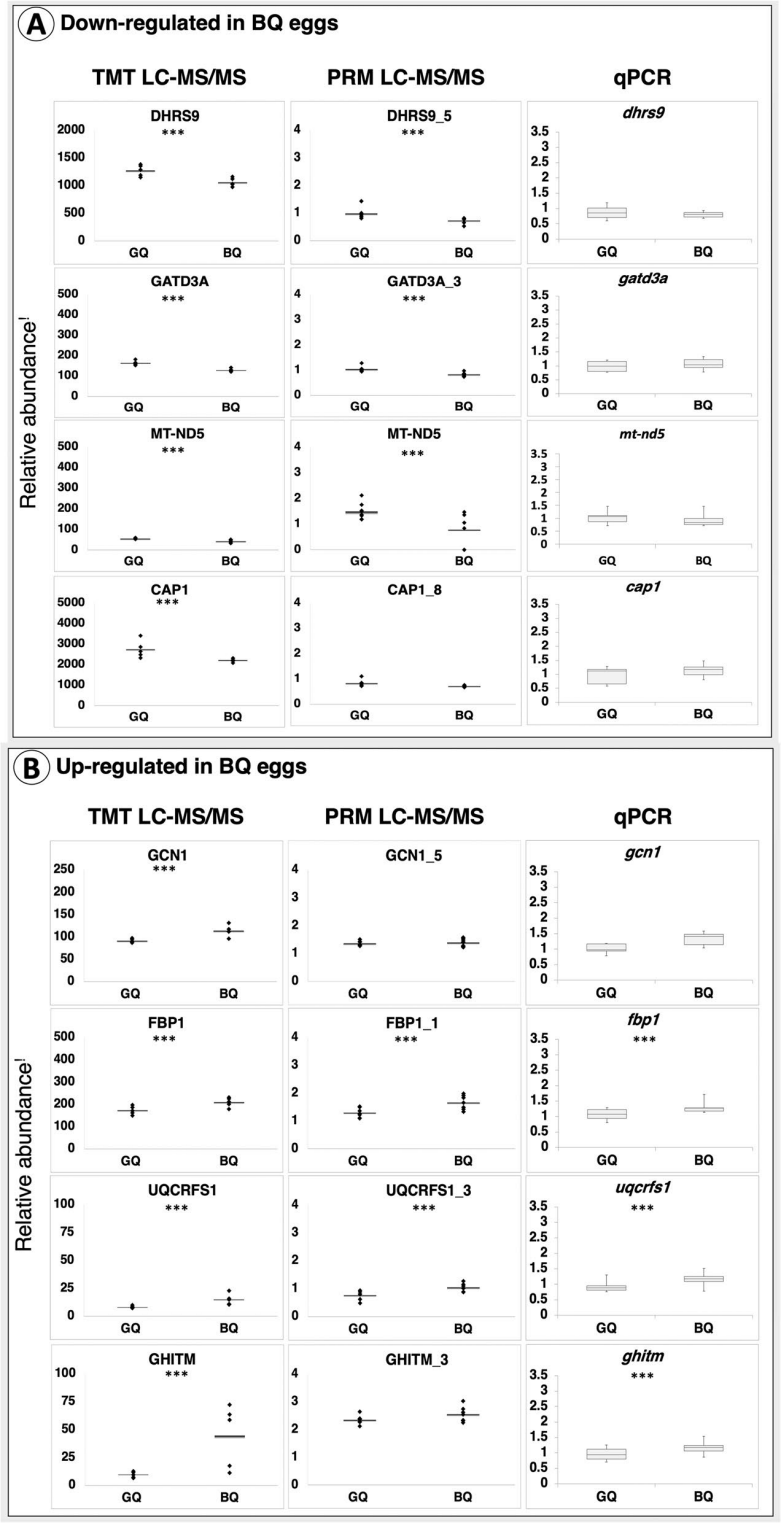
Comparison of marker protein abundance and corresponding gene expression. Panel **A** Proteins down-regulated in poor quality eggs Panel **B** Proteins up-regulated in poor quality eggs. Asterisks indicate statistically significant differences (*p* < 0.05). Relative abundance^!^ represents peak area intensities for protein abundances and gene copy numbers (normalized to transcript copy numbers of halibut *18S*) for gene transcript abundances. GQ: Good quality eggs BQ: poor quality eggs

Differential abundance of 8 of the 21 candidate marker proteins (MT-ND5, DHRS9, GATD3A, CAP1, GCN1, FBP1, UQCRFS1, GHITM) between good and poor quality eggs was confirmed by PRM-based LC-MS/MS (Fig. S4). The number of proteins targeted by this method was limited by the availability of peptides that were suitable for use as references for this study (see Material and Methods section for details). Results revealed all candidate marker proteins, except GHITM, to exhibit the same tendency of regulation relative to egg quality as was detected by TMT-labeling-based LC-MS/MS. However, only five candidate proteins (MT-ND5, DHRS9, GATD3A, FBP1, UQCRFS1) were found to significantly differ in abundance between good and poor quality eggs. Results were consistently stable for all representative heavy peptides, which varied from 1 to 3 in number of cases per candidate protein. Respectively, FBP1 and UQCRFS1 were up-regulated while MT-ND5, DHRS9 and GATD3A were down-regulated in poor quality eggs (Fig. S4). Comparisons of protein abundance quantified via TMT- versus PRM-based LC-MS/MS, and of gene expression quantified by qPCR, for the eight candidate marker proteins, are given in Fig. 5.

### Transmission electron microscopy and mtDNA levels

**Fig. 6:**
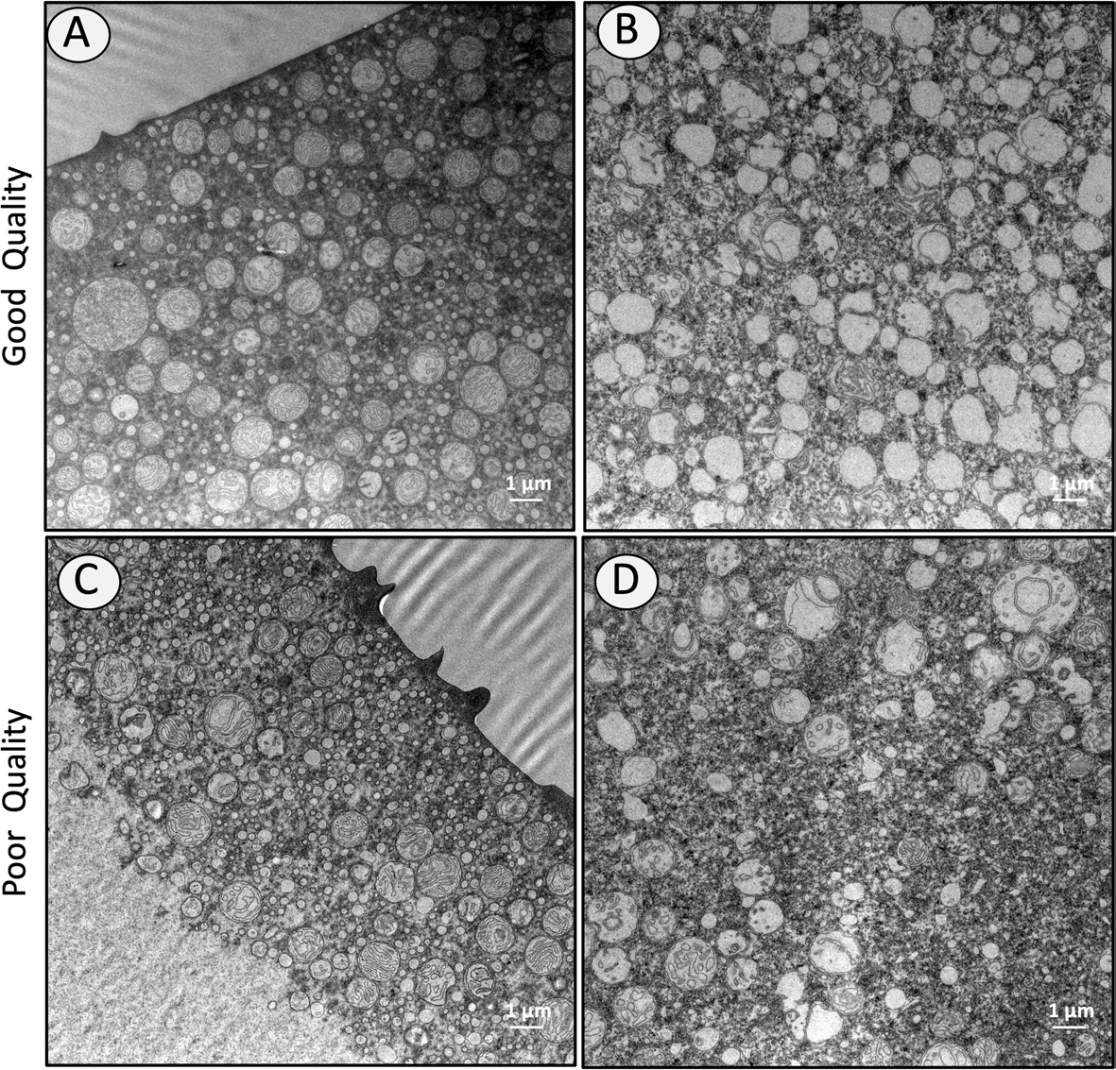
TEM images representing variability of observations between eggs from good and poor quality batches. Despite standard treatment of biological samples, a high variability in morphology was sometimes observed between eggs from the same batch. Panel **A** represents an egg containing a high number of well-formed mitochondria while Panel **B** represents an egg containing a high number of completely deformed mitochondria. Both eggs belonged to the same good quality batch and were kept in the same tube during fixation and postfixation. Panels **C** and **D** represent eggs containing variable numbers of better-shaped mitochondria. Both eggs belonging to the same poor quality batch and were similarly kept within the same tube during fixation and postfixation. All images depicted in this figure were generated by the authors for use in this article. Scale bars indicate 1 μm at 8 K magnification

Transmission electron microscopy (TEM) was conducted to detect possible differences in mitochondrial morphology or numbers between good and poor quality eggs. Number of vesicles with double membranes, as seen in intact mitochondria, and the number of intact mitochondria (those with ≥5 cristae) were significantly higher in poor quality eggs (*p* = 0.000724 and *p* = 0.010729, respectively) (Fig. S5). Poor quality eggs contained a ~ 1.3 x higher number of vesicles and a ~ 1.2 x higher number of intact mitochondria relative to good quality eggs. Poor quality eggs additionally exhibited a significantly higher (~ 1.3 x) number of cristae per mitochondria on average in comparison to good quality eggs (*p* = 9.21E-15). In contrast, good quality eggs contained larger, well-formed mitochondria with significantly higher mitochondrial area (μm2) and mitochondria circularity (*p* = 1.15E-08 and *p* = 0.016094, respectively) (Fig. S5). There was no significant difference between good and poor quality eggs in total mitochondrial area per unit of cytoplasmic area (*p* = 0.408). A high variation among females of the same quality group and within eggs from the same batch was observed. Some eggs from good quality batches contained irregularly-shaped, empty vesicles (possibly former mitochondria) while some others from poor quality batches exhibited well-formed mitochondria with well-defined cristae. Moreover, evidence of possible mitochondrial fusion was observed in both good and poor quality eggs (Fig. 6, Fig. S6).

**Fig. 7:**
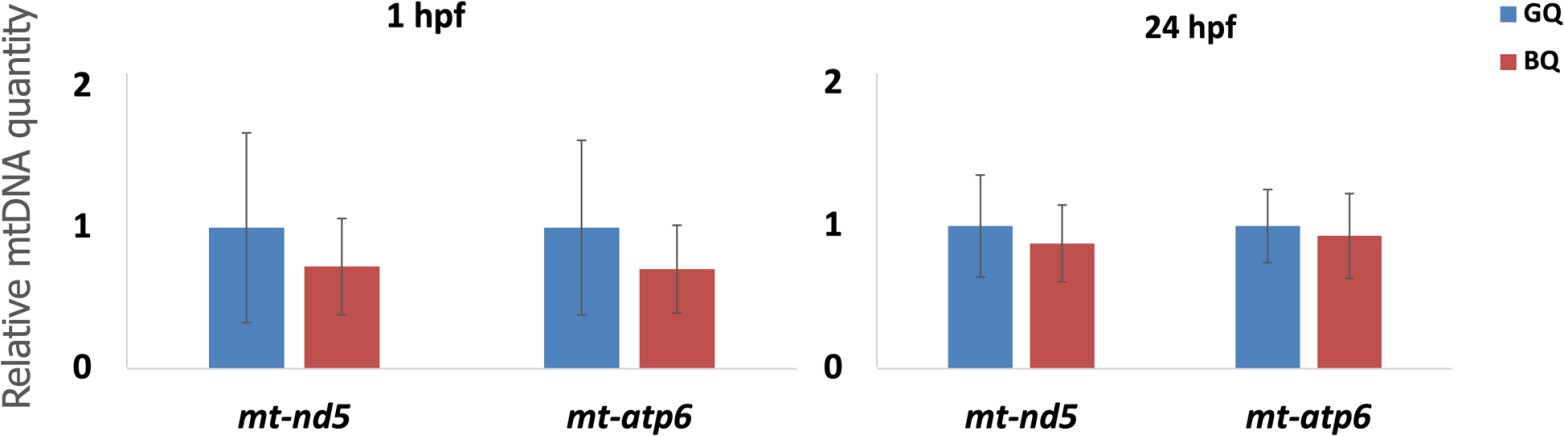
Mitochondrial DNA Quantification. Genomic DNA abundance for *mtnd5* and *mt-atp6* was measured via TaqMan qPCR using the standard curve method for eggs at 1 hpf and the DDCT method with 18S ribosomal RNA as reference gene for eggs at 24 hpf. Results indicate no statistically significant differences in abundance of mtDNA between good and poor quality eggs at either stage (*p* > 0.05). GQ: Good quality eggs BQ: poor quality eggs

The higher incidence of small and poorly formed mitochondria containing higher numbers of cristae in poor quality halibut eggs led us to quantify and compare genomic mitochondrial DNA levels (*mt-nd5* and *mt-atp6*) in good versus poor quality eggs. Results did not reveal any statistically significant differences in mtDNA levels at 1 hpf or 24 hpf stages (*p* > 0.05) (Fig. 7).

## Discussion

### Overview of the biological status of Atlantic halibut eggs

The present study was carried out to gain insight into the molecular mechanisms involved in egg quality determination in Atlantic halibut. The 1-cell-stage embryo was chosen as the biological material for laboratory analyses in order to allow comparisons with previous results obtained from zebrafish [22, 24]. Females of diverse backgrounds (source, time in captivity, age, size, reproductive experience, and prior performance) were used as egg donors to promote coverage of multiple factors potentially involved in egg quality determination. Female reproductive fitness and egg quality are deeply influenced by many internal elements (i.e. age, stress tolerance, nutritional status, spawning experience, domestication, etc.) and external factors (temperature, photoperiod, etc.) [4, 5]. While halibut females may release eggs into the tank, spawning behavior is rarely present in captivity and in order to obtain eggs of high quality, broodstock females must be followed individually and stripped as close to ovulation as possible [26]. Based on our experience, Atlantic halibut that have been in the system for over 15 years tend to produce low quality eggs and may be reproductively senescent (Møgster, Yilmaz and Norberg, unpublished observations). Females with little spawning experience and females newly introduced to spawning in captivity tend to have difficulty generating and releasing high quality eggs, indicating that both age and domestication are important factors for successful spawning in captivity.

An egg quality assessment protocol based on embryo survival prior to hatching was established based on our experience in hatchery practices which indicate stabilization in embryo survival percentage prior to hatching, around 12 dpf (Møgster, Yilmaz and Norberg, unpublished observations). However, correlation assessments between all measured reproductive parameters (female fecundity, egg buoyancy, fertilization rate, normal cell division, survival prior hatching) and survival rate were also made (see Fig. S1). In contrast to some other marine species spawning pelagic eggs, Atlantic halibut show no clear relation of egg buoyancy to embryo survival. The highly significant and strong correlation between egg fertilization rate and normal cell division to egg quality makes it possible to use these parameters to preselect egg batches for incubation. However, some egg batches with high fertility and rates of progress through early cleavage stages fail substantially later in development. This inconsistency in practice sets forth the offspring survival rate as the most reliable parameter to be used in egg quality assessment in halibut. Biological samples were collected for analyses over three consecutive reproductive seasons (2019–2021) with limited changes to the set of female spawners, allowing confirmation of the precision of our egg quality assessments and findings at proteomic and transcriptomic levels. The close cut off for survival rate differences between batches of good and poor quality eggs (GQ ≥72 BQ ≤71) ensured sensitivity of our findings.

### Protein homeostasis

Cellular functions during embryogenesis rely on proteostasis, defined mainly by the appropriate regulation of protein synthesis, folding, and degradation [27]. The precise level of protein synthesis ongoing in early stages of embryonic development in fishes is unknown. However, correct protein translation and folding is a crucial step in protein synthesis since accumulation of misfolded and/or unfolded proteins in the ER lumen disturbs its functioning, leading to ER stress which can have severe consequences for developmental competence.

Overall observations from this study suggest that normal protein translation and folding activities are partially blocked or otherwise abnormal in poor quality halibut eggs. Accordingly, the results of global proteomic profiling indicate impaired proteostasis in halibut eggs of poor quality. Examples include the higher frequency distribution of proteins related to protein translation and folding in good quality eggs but of proteins related to transcription and protein degradation and synthesis inhibition in poor quality eggs. Additional results on overrepresentation of proteins related to protein folding, RNP complex biogenesis, and RNA catabolic processes in good quality eggs in contrast to overrepresentation of proteins related to RNA splicing and mRNA processing indicate the presence of ER stress conditions and activated UPR mechanisms in poor quality eggs. The absence of a closely interlinked protein homeostasis network compared to that seen in good quality eggs, and the down-regulation of proteins related to protein synthesis (PDIA4, PPID, GFM), in poor quality eggs strengthen this hypothesis. These findings strikingly resemble those reported for poor quality zebrafish eggs [22] and for eggs from zebrafish lacking functional type I and type III vitellogenin genes [24]. Even though a strong connection between proper function of the multiple Vtg system and egg quality is yet to be established [28, 29], these common proteomic signatures of poor egg quality present in two evolutionary distant species underscore the need for investigations targeting their association with egg quality in a diverse assemblage of fishes in future studies.

Despite the need for more detailed studies of ER stress signaling and UPR activation, our present findings are a first step toward understanding potential impairments of these mechanisms in poor quality fish eggs. Heat, osmotic and pH stress, maternal nutrition and physiology, ovarian oxidative stress, oxygen and glucose availability and limitations in fatty acid availability are among the factors known to induce ER stress and activation of UPR and ER stress signaling in oocytes and embryos of several mammals, including mice, pigs, bovine species, rabbits and humans [30]. The multifarious backgrounds of female spawners in the present study makes the homogeneity of findings within egg quality groups remarkable, but it also makes it difficult to infer the proximal causes of the identified impairments. Further research in this area is clearly warranted, particularly with respect to when in oogenesis and under what conditions the hallmarks of poor egg quality arise.

### Energy homeostasis and mitochondrial biogenesis

Biological activities supporting cell division in newly fertilized eggs of oviparous animals are mainly dependent on maternal transcripts, proteins, lipids, and other key molecules loaded into the oocyte prior to final maturation and ovulation. The high amount of energy required to conduct these activities is mainly provided by a normally functioning pool of mitochondria, which is produced during early oogenesis and peaks during late stages of folliculogenesis [31, 32]. Before embryonic mitochondria take over, the embryo is dependent on the functioning of the existing cohort of maternal mitochondria to provide the energy required for viability and development [33, 34]. Deficiencies in mitochondrial structure and function have been shown to impact egg quality and developmental competence in a broad array of species including humans [32, 35].

The overrepresentation of proteins involved in mitochondrial biogenesis and energy metabolism (see Fig. 2), enrichment of GO Biological processes and Cellular component terms with mitochondrial functions and molecular structures, respectively, and of KEGG pathways with mitochondrial activities (see Fig. 3), in addition to resolution of the specific STRING subnetwork mainly formed by proteins related to mitochondrial biogenesis, organization and energy homeostasis (see Fig. 4) were taken together as strong indicators of aberrant mitochondrial processes in poor quality eggs in this study. Additional results indicating quantitative differences in the abundance of several proteins related to mitochondrial biogenesis and energy homeostasis (see Fig. 5) between good and poor quality halibut eggs (e.g. MT-ND5, GATD3A, PHB, ACLY, CYC1, FH, UQCRB, GHITM, UQCRFS1, FBP1, and ATP5F1B) led us to further evaluate their potential as candidate egg quality markers at both proteomic and transcriptomic levels. Interestingly enough, six of these candidate marker proteins (UQCRB, CYC1, UQCRFS1, ATP5F1B, FH, FBP1) were found to fall into the STRING network formed by proteins up-regulated in poor quality eggs. Gene expression levels for all these proteins, and more, could be quantified successfully by qPCR, while the availability of appropriate target peptides to be used in PRM-based LC-MS/MS limited the number of proteins that could be investigated at this level. Nevertheless, validation of our findings in egg sample sets collected over three reproductive seasons for five of these candidate egg quality marker proteins (MT-ND5, GATD3A, GHITM, UQCRFS1, FBP1) was accomplished successfully via both PRM-based LC-MS/MS and qPCR methodologies.

Measurements of abundance of these several mitochondrial proteins and of their corresponding gene transcripts revealed significant differences between good and poor quality eggs. The non-significantly lower average protein and transcript abundances of MT-ND5 and MT-ATP6, in contrast to significantly higher protein and transcript abundances of CYC1, UQCRB, UQCRFS1 and ATP5F1B, seen in poor quality eggs is intriguing, considering that they are all key components of the inner mitochondrial membrane. The disparate expression of these proteins and of their corresponding genes may be considered as indicators of structural and functional impairments in mitochondria.

Highly significant overrepresentation of the amino acid degradation, fatty acid degradation, and glycolysis/gluconeogenesis KEGG pathways in good quality eggs, in contrast to overrepresentation of several human neurodegenerative disease pathways and mitochondrial function related pathways in poor quality eggs may indicate potential for more than one problem at the mitochondrial level, perhaps including lack of substrates for mitochondria to generate energy in addition to structural deficiencies. Causes and factors leading to these potential problems are largely unknown and will require additional research to be discovered. The prominent KEGG pathways in poor quality eggs revealed by network enrichment analyses, such as Alzheimer’s disease, Parkinson’s disease, Huntington’s disease, oxidative phosphorylation, and citrate cycle in addition to cardiac muscle contraction are highly consistent with the findings from *vtg* knock out zebrafish eggs, which emulated poor quality eggs in this regard [24]. All of these pathways seem to be interconnected [36–40] and have been previously reported to be linked to perturbations in mitochondrial maintenance, localization, and activity along with aberrant protein folding, leading to subsequent impairment of normal development [39, 41]. The cardiac muscle contraction pathway was previously linked to a cardiac and yolk sac edema phenotype observed in *vtg*-KO zebrafish eggs [23, 24]. Further studies targeting morphological observations of development by offspring originating from different quality batches of halibut eggs are needed.

The clear linkage of mitochondrial proteomics to halibut egg quality discussed above led us to evaluate mitochondrial abundance and morphology by transmission electron microscopy (TEM). Two parameters considered to represent mitochondrial abundance were 1) the number of vesicles containing double membranes that resemble mitochondria, and 2) the number of typical mitochondria (containing ≥5 apparent cristae) per unit of cytoplasmic area. Both parameters were found to be consistently and significantly higher in poor quality eggs. In addition, the number of cristae per mitochondria was also significantly higher in poor quality eggs (see Fig. S5). These results appear controversial considering the many prior observations that low numbers of mitochondria, and also low copy numbers of mtDNA, are indicators of low oocyte quality and poor embryonic developmental competence in several organisms [13, 34, 42–44]. However, they are in accordance with findings from other studies contradicting the utility of mtDNA copy number as a marker for embryonic competence in humans [44–48]. To test for potential relationships between mtDNA levels, mitochondrial abundance and egg quality we quantified genomic DNA levels of two key mitochondrial genes (*mt-nd5* and *mt-atp6*). Results revealed no statistically significant differences in mtDNA abundances between good and poor quality eggs at both the 1 hpf and 24 hpf stages. These findings were in accordance with those from a previous study on transcriptome analysis of egg viability in rainbow trout, *Oncorhynchus mykiss* [13]. In apparent contrast with the non-significant differences in DNA and transcript abundances of *mt-nd5* and *mt-atp6*, the significantly higher transcript and protein abundance for some other mitochondrial proteins is intriguing. Significantly higher transcription activities resulting in high numbers of malformed mitochondria despite the similar mtDNA abundance in poor quality eggs might indicate impairments at gene expression and protein synthesis levels. An increasing number of mitochondria has been proposed to be linked to compensatory response of the cell to mitochondrial mutations leading to impaired function and reduction in energy synthesis [49]. The smaller and more irregularly shaped mitochondria seen in poor quality halibut eggs provide supportive evidence of potential structural deformities which might be related to ER stress and protein folding deficiencies. The overall results of TEM in this study are consistent with, and complementary to, the proteomic and transcriptomic findings. However, high variability between females of the same quality group and within eggs from the same female necessitate extension of this study with a higher number of egg quality replicates.

## Conclusions

This study provides concrete evidence of signatures of impairments in protein and energy homeostasis in newly fertilized, poor quality Atlantic halibut eggs. These critical impairments and subsequent cellular dysfunctions are evidenced by highly consistent and complementary results from global proteomic profiling, next-generation targeted proteomics, direct measurements of transcript and mtDNA abundance and TEM observations in egg samples collected during three sequential reproductive seasons. The highly variable background of female halibut donors of disparate quality egg batches in this study strengthens the legitimacy of the observed molecular signatures indicating that they are hallmarks of egg quality in this species. Moreover, consistency between findings from this study and previous research on zebrafish suggest a common stereotypical sequence of interconnected events leading to defective developmental competence among fishes, and possibly other vertebrates. Additional research is needed to discover when and under what conditions these defects may arise, and to what extent they are observed in poor quality eggs of other species. This study sets the stage for progress in these areas, advancing our fundamental understanding of the molecular basis of egg quality and embryonic developmental competence.

## Methods

Figs S7 and S8 summarize the process of sample collection and the implemented experimental design, respectively.

### Animal care and sample collection

Egg samples from *N* = 10, 8, and 6 batches of Atlantic halibut were collected in 2019, 2020 and 2021 reproductive seasons, respectively. Collected samples were from females with various background, The pool included young and aged females (~ 8 to ≥17 yrs), small and large females (25–70 kg and 110–167 cm), females originated from the wild and F1 generation which were bred in captivity, females which were newly introduced to the system and those with experience in the system (3 and 12 yrs., respectively), and finally females which interchanged in the quality of eggs they released from year to year and those consistently spawning good or poor quality egg batches every year.

A total of 13 mature female and 6 male halibut (wild captured or produced in captivity at the Austevoll Research Station of Institute of Marine Research in Norway) were kept in 7 m diameter (50 m^3^ capacity) circular tanks at the same facilities with simulated natural daylight conditions and sea water at salinity of 34 ppt, taken from 160 m depth. Water temperature ranged from 7.8 to 9 °C from May to December, and was constant at 6 °C until the end of the spawning season. Fish were hand-fed with an artificial broodstock diet (VITALIS Cal 22 mm, Skretting, Norway) every other day to satiety, except during the spawning season when appetite was low (February–May).

Females were followed closely at the start of the spawning season to determine the first egg release time point and following that were checked every 36–42 h for the onset of the following batch release based on morphological changes in the abdominal region. Eggs from spawns between the 3rd and the 5th batch were targeted in this study to ensure the fine tuning of spawning rhythm and stability of egg quality during the season in each female. Following the predicted ovulation of the targeted batch, which occurs at approximately 72–92 h after the release of the previous batch, eggs were stripped from mature females and fertilized with sperm collected immediately after. Replicates of 0.5 ml eggs per spawn were snap frozen in liquid nitrogen at 1-cell stage after fertilization and stored at − 80 °C until analysis (Fig. S7). However, the In addition to their large size, our broodstock were not anesthetized during sample collection out of concern for possible negative effects of the anesthetic over time on eggs developing in the following batches. High expertise and long-term experience of our technical personnel in gamete collection from halibut not treated with an anesthetic allowed for a quick and humane handling of the animals. Following the sample collection, both female and male halibut were kept at our station as broodstock for production of biological material for future research. Ethical permissions for the practice have been obtained from Norwegian authorities and the related protocol numbers are stated at the Ethical concerns section.

Hundred milliliters (~ 3500–4000) of fertilized eggs from each spawn were incubated in 250 l incubators and were kept in darkness, at 6 °C until hatching. Daily care involved removal of dead embryos from the bottom of incubators and measurement of their volume for mortality determination. Egg quality assessments were based on embryo survival prior to hatching at 12 dpf (days postfertilization). Embryos used in this study were euthanized with anesthetic overdose of Finquel vet (200 mg/l) at the end of the experimental period. Information on other parameters such as fecundity, fertilization rate, normal cell division, egg buoyancy was also collected to test for potential correlation with egg quality. Fecundity indicates the volume of eggs (in l) collected per batch. Fertilization percentage indicates the ratio of embryo with successful visible cell division at 24 hpf. Normal cell division percentage represents the number of embryos with symmetrical cell division at 24 hpf. Buoyancy indicates egg densities (g/cm^3^) measured at a buoyancy column at 1 hpf. Egg batches with embryonic survival rates of ≥72 were considered to be of good quality and those spawns with ≤71 embryonic survival were considered to be of poor quality. The list of egg batches collected during each year and egg quality assessment parameters and classifications are given in Table S1.

### TMT labeling based LC-MS/MS

Egg samples (0.5 ml) from a total of 10 spawns (*N* = 5 spawns for good quality, *N* = 5 spawns for poor quality) collected during the 2019 reproductive season were lysed in 1 ml modified RIPA lysis buffer (pH 7.4) containing; 50 mM Tris, 150 mM NaCl, 1% NP-40, 1% SDS, 1% CHAPS, 0.5% SDC, 1x protease inhibitor cocktail (cOmplete™ ULTRA Tablets, Roche). Sample lysis, protein concentration measurement and sample reduction processes were carried out as indicated by [50] with the following modifications: Samples were sonicated in 6 steps of 30 sec at 40% amplitude with 30 sec stops between each followed by 30 min incubation on ice and centrifugation at 16200 x g for 30 min at + 4 °C. Protein extracts containing 30 μg of total egg proteins were diluted with lysis buffer to 40 μl total volume and reduced with 4 μl of 100 mM DTT for 1 h at room temperature (RT) (10 mM of final concentration). Samples were then alkylated with 6 μl of 200 mM Iodoacetamide (IAA) by incubation in dark for 1 h at RT. Alkylated samples were then enhanced using Single-Pot Solid-Phase-enhanced Sample Preparation (SP3) according to the protocol by [51]. Mix of two types of Sera-Mag SpeedBeads 50 mg/ml (GE Healthcare) was prepared at 75 μg/μl bead concentration in 47 μl of water. Four μl of beads mix at a bead/protein ratio of 10:1 (wt/wt) were added onto each alkylated sample along with 126 μl of 100% ethanol (to 70% final ethanol concentration). Following 7 min incubation on a thermomixer at 1000 rpm 24 °C samples were washed 3 times in 80% ethanol. A MagRack system was used to facilitate removal of liquid without disturbing the beads containing proteins of interest.

Tryptic digestion of proteins was carried out using porcine trypsin (Promega, GmbH, Mannheim, Germany). Trypsin solution prepared in 100 mM Ambic and 1 mM CaCl_2_ at a 0.01 μg/μl concentration and 100 μl added onto each sample (trypsin to sample ratio 1:25). Samples containing trypsin were then sonicated twice for 30 sec and incubated for 16 h at 1000 rpm 37 °C. Peptides were then recovered by centrifugation at 13000 rpm for 3 min at RT. A second recovery was performed by washing beads with 0.5 M NaCl via pipetting and 2 x ultrasound sonication for 30 sec and centrifugation at 13000 rpm for 3 min at RT. Second recovery of peptide digests was combined with the previous one and peptide concentration was determined on Nanodrop to check for sufficient recovery. Peptide mixtures were desalted and concentrated on reverse-phase Oasis HLB μElution Plate (Waters Corporation, Manchester, UK) as indicated by [52]. Lyophilized peptides mixtures were reconstituted in 52 μl of 100 mM Triethyl ammonium bicarbonate (TEAB) buffer and peptide concentrations were determined on Nanodrop to check that peptide amounts were equal in all samples prior to labeling using TMT10plex™ Isobaric Label Reagent Set, 1 × 0.8 mg (ThermoFisher Scientific). Twenty-one μl of each label (~ 0.4 mg) were added onto 50 μl of samples containing ~ 20 μg of peptide digests. After 1 h incubation at RT, 4 μl of 5% Hydroxylamine (NH_2_OH) were added and samples were incubated for an additional 15 min at RT to quench the reaction. All ten vials of samples were combined and approximately 100 μg peptide digests from this mix were fractionated using Pierce High pH Reversed-Phase Peptide Fractionation Kit (ThermoFisher Scientific) according to instructions from the manufacturer. All fractions were lyophilized and reconstituted in a mix of 0.5% Formic acid (FA) and 2% ACN (at ~ 0.5 μg/μl concentration) prior injection. Samples were then desalted and loaded on an LC-MS/MS system as indicated by Bjørlykke et al. [53] with the following modifications. The gradient composition was 5% B during trapping (5 min) followed by 5–7% B over 0.5 min, 7–22% B for the next 59.5 min, 22–35% B over 22 min, and 35–80% B over 5 min. Elution of very hydrophobic peptides and conditioning of the column were performed during 10 min isocratic elution with 80% B and 15 min isocratic conditioning with 5% B, respectively.

MS spectra acquisition resolution R was 60,000 at m/z 200, and the maximum injection time was 50 ms. The 12 most intense eluting peptides above the specific intensity and charge states indicated by Bjørlykke et al. [53] were sequentially isolated prior fragmentation. Fragmentation was performed with a normalized collision energy of 32%, and fragments were detected in the Orbitrap at a resolution of 60,000 at m/z 200, with first mass fixed at m/z 110. One MS/MS spectrum of a precursor mass was allowed before dynamic exclusion for 30 sec with “exclude isotopes” on. Lock-mass internal calibration (m/z 445.12003) was used. The spray and ion-source parameters were as follows. Ion spray voltage of 1800 V, no sheath and auxiliary gas flow, and a capillary temperature of 275 °C conditions were additionally set for data acquisition.

### Data search

Obtained spectra searched against an in-house built proteome database originated from halibut egg transcriptome with additional peptide sequences for mitochondrial proteome and the vitellogenin proteins from this species. See *Availability of data and materials* section for accessibility of the mitochondrial genome database. Data search was performed using the Sequest HT search engine implemented in Proteome Discoverer 2.4 (Thermo Fisher Scientific). Trypsin was selected as protease with a maximum of two missed cleavage sites and cysteine carbamidomethylation and TMT10plex mass tags both at peptide N-terminus and Lysine side chain as fixed modifications. Methionine oxidation was selected as variable modification with a maximum of three such modifications per peptide. The precursor mass tolerance threshold was 10 ppm and the maximum fragment mass error 0.02 Da. A signal-to-noise filter of 1.5 was applied for precursor ions, and only charge states from two to five were used in the search. Filtering out the false positive peptide identifications were performed by means of False Discovery Rate (FDR) on the reversed database, estimated using the Percolator algorithm (http://per-colator.com). Peptide hits were filtered for an FDR of q < 0.01. In addition to the FDR filter, high confident threshold score filters for Sequest HT (cross correlation scores, XCorr) were as follows: 1.9 (z = 2), 2.3 (z = 3), 2.6 (z = 4 or higher). Only proteins/protein groups that were identified by two or more independent peptide hits were accepted as true positive identifications. Proteins that contained similar peptides and could not be differentiated based on MS/MS analysis alone were considered an equivalence class by using the protein grouping algorithm. Only master proteins from each group were considered for the following quantification analysis. Common laboratory contaminants (keratin and albumin proteins) were removed prior to following analysis. The mass spectrometry proteomics data have been deposited to the ProteomeXchange Consortium via the PRIDE [54] partner repository with the dataset identifier PXD029894 and a project DOI number of 10.6019/PXD029894 and is publicly available.

### Data analysis

Detected proteins were mapped against a common database for all organisms with available correspondent sequences and were identified based on their identities. Protein abundances were quantified based on peak area intensities. Accordingly, differentially abundant proteins were determined based on *p* values resolved from independent samples t-test (*p* < 0.05) followed by Benjamini Hochberg correction for multiple testing (*p* < 0.05) using the SPSS software (IBM SPSS Statistics Version 19.0.0, Armonk, NY). Functional annotation of proteins found to be differentially abundant between good and poor quality eggs was performed using the UNIPROT and KEGG functional annotation tools. These proteins were then classified into thirteen arbitrarily chosen functional categories that would account for > 90% of the proteins as originally suggested by [22] with slight modifications. These functional categories are: transcription, translation, protein folding, protein transport, energy metabolism, mitochondrial biogenesis, cell cycle, division, growth and fate, lipid metabolism, metabolism of cofactors and vitamins, protein degradation and synthesis inhibition, oxidoreductase (redox)- and detoxification (detox)-related, and immune response-related [22]. Significant differences between groups in the distribution of differentially regulated proteins among functional categories detected using Chi square analysis (*p* ≤ 0.05). A list of these proteins, along with the NCBI gene IDs, NCBI accession numbers, associated protein names from human database, protein full names, functional categories, significance of differences in abundance, relative abundance ratios, and regulation tendencies are given in Table S2 (See *Availability of data and materials* section for details of used reference databases).

Gene ontology overrepresentation analyses were conducted using the GESTALT (WEB-based GEne SeT AnaLysis Toolkit) [55] available online at for Biological Process, Molecular Function, and Cellular Components, and KEGG Pathway terms using human proteins as reference database. Proteins which were differentially regulated between good and poor quality halibut eggs were additionally subjected to the analysis of protein-protein interaction networks [56, 57] separately using the STRING Network search tool available from the STRING Consortium online at https://string-db.org/cgi/input?sessionId=b1QVfHtqmBW4&input_page_active_form=multiple_identifiers with the data settings Confidence: Medium (0.40), Max Number of Interactions to Show: None/query proteins only. See *Availability of data and materials* section for details of used reference databases. For the GESTALT and STRING analyses, only statistically significant enrichment results (*p* < 0.05) are reported.

### TaqMan based quantitative real time PCR

Gene expression for a total of 21 proteins were tested in good versus poor quality halibut eggs using TaqMan based quantitative real-time PCR (qPCR). Total RNA extraction from frozen *N* = 19 egg batches, collected from 2019 and 2020 seasons, was performed using TRI Reagent™ (Thermo Fisher Scientific). cDNA was synthesized using SuperScript™ VILO™ cDNA Synthesis Kit (Thermo Fisher Scientific) from 1 μg of DNAse treated (DNase I, Amplification Grade, Thermo Fisher Scientific) total RNA with 260/280 absorbance ratios of 1.9–2.1 (Nanodrop Spectrophotometer, Thermo Fisher Scientific) and RNA integrity values of 9–10 (Bioanalyzer, Agilent Technologies). Gene-specific primers and dual-labelled probes (labelled with 6-carboxyfluorescein and BHQ-1, Black Hole Quencher 1 on 5′ and 3′ terminus, respectively) were designed using Eurofins Genomics qPCR assay design tool available online at https://eurofinsgenomics.eu/en/ecom/tools/qpcr-assay-design/ and Integrated DNA Technologies (IDT) PrimerQuest Tool available online at https://eu.idtdna.com/Primerquest/Home/Index. Gene sequences obtained from the halibut mitochondrial transcriptome generated from mitochondrial genome available at https://www.ncbi.nlm.nih.gov/nuccore/CM020214.1?report=fasta (See *Availability of data and materials* section for details). Designed primers were additionally analyzed for secondary structures using IDT Oligo analyzer tool available online at https://eu.idtdna.com/calc/analyzer and produced by Eurofins Genomics. Sequences of these primers and probes used in this experiment are given in Table S4.

Each qPCR was performed in triplicates of 10 μl reactions containing cDNA (diluted at 1:100), 400 nM of each primer, 200 nM of hydrolysis probe, and 1x TaqMan Fast Advanced Master Mix (Applied Biosystems, Thermo Fisher Scientific) according to the manufacturer’s instructions in optical plates on a QuantStudio 5 Real-Time PCR system (ThermoFisher Scientific) equipped with 384-well block. No-template controls for each gene were included for each assay. PCR cycling conditions were as follows: 50 °C for 2 mins, 95 °C for 20 s, 40 cycles at 95 °C for 1 s followed by an annealing-extension at 60 °C for 20 s. The gene expression abundance within a sample set, relative to Atlantic halibut18S, was calculated using the 2^−ΔΔCt^ mean relative quantification method in this study. Obtained data were subjected to independent samples t-test, *p* < 0.05) followed by Benjamini Hochberg correction for multiple tests, *p* < 0.05 (IBM SPSS Statistics Version 19.0.0, Armonk, NY).

### Parallel reaction monitoring based LC-MS/MS

Eight out of a total 21 proteins (MT-ND5, CAP1, DHRS9, GCN1, GHITM, GATD3A, FBP1, UQCRFS1), which were previously determined as differentially abundant between good and poor quality egg batches using the TMT labeling based LC-MS/MS methodology, were carried out for further assessments as potential candidate biomarkers of egg quality in halibut. A parallel reaction monitoring based LC-MS/MS approach was followed in order to validate the differential abundance of these proteins between good and poor quality eggs originated from spawns collected both in 2019 (*N* = 4 spawns for good quality, *N* = 4 spawns for poor quality) and in 2020 (*N* = 4 spawns for good quality, *N* = 4 spawns for poor quality). Egg samples were processed in the same manner as mentioned above for TMT labeling method until prior to isobaric labeling step. About 2–3 target peptides were selected for each protein based on the following criteria collected from [58–61]; uniqueness to the target protein, length of 5–26 aa, ~ 50% hydrophobicity, no PTMs, no missed cleavages, positioned far downstream from N- or upstream from C-terminal, proper fragmentation (more than 3–4 fragment ions with well-defined peaks), peptide spectral matches (PSMs) (min 3), charges (min 2–3) and clear clustering in peptide abundance between good and poor quality eggs (Fig. S9). Peptide PRM compatibility and hydrophobicity tests were performed using Peptide Synthesis and Proteotypic Peptide Analyzing Tool available online at ThermoFisher Scientific. List of target proteins and their corresponding target peptides are listed in Table S5. Target peptides for each of these proteins were purchased in stable isotope labelled synthetic peptides (SIS) form in crude quality from Thermo Scientific. The C-terminal lysine or arginine in the SIS peptides were replaced by isotope labelled lysine (^13^C_6_, ^15^N_2_) or arginine (^13^C_6_, ^15^N_4_), resulting in a mass difference of 8 Da and 10 Da, respectively, to the corresponding endogenous peptide. The SIS peptides were spiked in equal amounts into the digested protein samples, at approximately the same level as the endogenous peptide, prior to desalting with Oasis HLB μElution Plate (Waters). The PRM data was analyzed using Skyline v1.4 [62] with the most abundant transition for quantification. Independent samples t-test was used to detect significant differences in abundance between good and poor quality eggs (*p* < 0.05).

### Transmission electron microscopy

Four to five eggs from each egg batch (*N* = 6 batches) that was collected during the 2021 reproductive season were prefixed in Karnovsky’s fixative [63] containing 5% glutaraldehyde, 2% paraformaldehyde, and 0.1 M Sodium cacodylate buffer for 24 h to allow fixation of the chorion to facilitate its mechanical removal. Dechorionated egg samples were placed back into Karnovsky’s fixative and transferred to the TEM facility for the consecutive steps of the sample preparation process. Eggs were postfixed in 1% osmium tetroxide (EMS # 19134) diluted in 0.1 M sodium cacodylate buffer on ice for 1 hour. Samples were then washed in buffer and dehydrated using a graded ethanol series (30, 50, 70, 96 and 100%) before being transferred to a 1:1 solution of 100% ethanol:propylene oxide in which they were incubated for 15 min. Samples were then incubated in 100% propylene oxide for 15 min before gradually introducing agar 100 resin (AgarScientific R1031)0. Samples were then incubated in a drop of 100% resin overnight and then placed in molds with fresh 100% resin at 60 °C for 48 h to polymerize. Ultrathin sections of approximately 60 nm were collected from *N* = 5 different regions of each egg representing good or poor quality batches. Images of ultrathin sections were taken at 8 and 20 K magnifications using Hitachi HT7800 transmission electron microscope with a 0.20 nm resolution lens (Off- axis, 100 kV) Emsis Xarosa (20 Mpix) bottom mounted CMOS camera (Model B20T) and the RADIUS 2.0 Software. Images at 8 K magnification were used to assess the number of vesicles with double membranes (see Fig. 6 and Fig. S6 for examples) which highly resembles intact mitochondria and the number of intact mitochondria (those with ≥ 5 cristae) per cytoplasm area. Images at 20 K magnification were used to assess the morphological differences such as the mitochondrial area (μm2), total mitochondrial area per cytoplasm area (μm2), mitochondria circularity and cristae number per mitochondria in a total of 1200 μm^2^ area for each egg. Mitochondria circularity is calculated as; 4π(Area)/(Perimeter^2), where 1.0 indicates a perfect circle, while 0.0 indicates an elongated shape. A minimum of 50 counts per egg were collected for the cristae number assessment. Independent samples t-test was used to detect significant differences in mitochondrial counts between good and poor quality eggs (*p* < 0.05) using SPSS (IBM SPSS Statistics Version 19.0.0, Armonk, NY).

### Mitochondrial gene quantification by real-time quantitative PCR

Relative abundance of genomic DNA for *mtnd5* and *mt-atp6* was measured via TaqMan qPCR using standard curve method in 1 hpf and DDCT method in 24 hpf halibut eggs. Serial dilutions of a single good quality sample with known DNA concentration were used as a reference for the standard curve method and the 18S ribosomal RNA was used as a reference for relative quantification using DDCT method. For gDNA extraction from 1 hpf eggs the insoluble materials leftover following homogenization in TRI Reagent during RNA isolation was mixed with 300 μl of 100% ethanol, tubes were inverted several times and incubated for 3 mins for genomic DNA isolation. The supernatant was removed after centrifugation at 2000 x g at + 4 °C and pellets were resuspended in 1 ml of 0.1 M sodium citrate in 10% ethanol (pH 8.5). Samples were incubated for 30 mins at RT mixing occasionally by gentle inversion. The supernatant was discarded after centrifugation for 5 mins at 2000 x g at + 4 °C, pellets were resuspended in 1.5 ml 75% ethanol and incubated for 20 mins by occasionally mixing by gentle inversion. Following centrifugation for 5 mins at 2000 x g at + 4 °C pellets were air dried for 5 mins and resuspended in 100 μl of water. gDNA extractions from 24 hpf eggs were performed using QIAamp DNA Mini Kit (Qiagen) following the instructions from the manufacturer. DNA concentrations were quantified using a Nanodrop Spectrophotometer (Thermo Fisher Scientific) and each qPCR reaction was performed in triplicates of 10 μl reactions containing 10 ng gDNA for 1 hpf and 40 ng for 24 hpf eggs, 400 nM of each primer, 200 nM of hydrolysis probe, and 1x TaqMan Fast Advanced Master Mix (Applied Biosystems, Thermo Fisher Scientific) according to the manufacturer’s instructions in optical plates on a QuantStudio 5 Real-Time PCR system (ThermoFisher Scientific) equipped with 384-well block. No-template controls for each gene were included for each assay. PCR cycling conditions were as follows: 50 °C for 2 mins, 95 °C for 20 s, 40 cycles at 95 °C for 1 s followed by an annealing-extension at 60 °C for 20 s. Assay efficiencies were at 98%, Slope: -3.368, R^2^: 0.999 and 100%, Slope: -3.307, R^2^: 0.998 for *mt-nd5* and *mt-atp6*, respectively. Obtained data were subjected to independent samples t-test, *p* < 0.05 (IBM SPSS Statistics Version 19.0.0, Armonk, NY). Sequences for primers and probes used in these assays are given in Table S4.

## Supplementary Information

**Additional file 1: Fig. S1.** Correlation of reproductive parameters with embryo survival and egg quality in halibut. **Fig. S2.** TMT labeling based LC-MS/MS quantification for candidate marker proteins. **Fig. S3.** TaqMan qPCR based relative quantification of gene expression for candidate marker proteins. **Fig. S4.** PRM based LC-MS/MS quantification of protein abundances for candidate marker proteins. **Fig. S5.** Results of counts and measurements of some mitochondrial parameters based on TEM imaging. **Fig. S6.** Some examples of mitochondrial observations in halibut eggs. **Fig. S7.** Biological samples and egg quality assessment procedure used in this study. **Fig. S8.** Experimental design. **Fig. S9.** Quantification of heavy peptides selected for use in PRM based LC-MS/MS. **Table S1.** List of females used in this study. **Table S2.** Differentially abundant proteins between good and poor quality halibut eggs based on peak area intensities obtained from the TMT based LC-MS/MS. **Table S3.** STRING protein-protein interaction networks enrichment analysis. **Table S4.** List of primers and probes used for relative quantification of gene expression and mtDNA quantification using TaqMan based qPCR. **Table S5.** List of reference peptides used for PRM based LC-MS/MS.

## Data Availability

The in-house built proteome database and the Atlantic halibut egg transcriptome from which it was generated are not publicly available. The additional mitochondrial proteome that was used to build the proteome database (generated from the *Hippoglossus hippoglossus* isolate fHipHip1 mitochondrion whole genome shotgun sequence - accession # CM020214.1) is available at https://www.ncbi.nlm.nih.gov/nuccore/CM020214.1?report=fasta. The Atlantic halibut gene identities and protein accession numbers given in Table S2 are originated from *Hippoglossus hippoglossus* (assembly fHipHip1.pri) genome database, a product of BioProject: PRJNA562001 - Vertebrate Genomes Project, is available online at https://www.ncbi.nlm.nih.gov/genome?LinkName=bioproject_genome&from_uid=562001. Gene names used in Table S2 originated from the Human Gene Nomenclature Database (DOI ID: 10.1093/nar/gkaa980 and PubMed ID: 33152070) is available online at https://www.uniprot.org/uniprotkb?query=(database:HGNC)**.** The human database used as a reference for enrichment and protein-protein interactions network analyses is available online at https://string-db.org/cgi/download?sessionId=bwD9wTkG94TW&species_text=Homo+sapiens. The mass spectrometry proteomics data have been deposited to the ProteomeXchange Consortium via the PRIDE partner repository with the dataset identifier PXD029894 and a project DOI number of 10.6019/PXD029894. Data is publicly available at http://proteomecentral.proteomexchange.org/cgi/GetDataset.

## Abbreviations

TMT: Tandem mass tag labeling
LC-MS/MS: Lliquid chromatography tandem mass spectrometry
GQ: Good quality
BQ: Poor quality
PRM: Parallel reaction monitoring
TEM: Transmission electron microscopy
mtDNA: Mitochondrial DNA
mRNA: Messenger ribonucleic acid
Vtg: Vitellogenin
KO: Knock out
CRISPR/Cas9: Clustered regularly interspaced short palindromic repeats and CRISPR associated protein 9
hpf: Hours post fertilization
dpf: Days post fertilization
NCBI: National Center for Biotechnology Information
GO: Gene ontology
RNP: Ribonucleoprotein
OP: Organophosphate
KEGG: Kyoto Encyclopedia of Genes and Genomes
TCA cycle: Citric acid cycle
NAFLD: Non-alcoholic fatty liver disease
STRING: Search Tool for the Retrieval of Interacting Genes/Proteins
qPCR: Quantitative real-time PCR
RIPA buffer: Radioimmunoprecipitation assay buffer
SDS: Sodium dodecyl sulfate
CHAPS: 3-[(3-Cholamidopropyl) dimethylammonio]-1-propanesulfonate
SDC: Sodium deoxycholate
DTT: Dithiothreitol
IAA: Iodoacetamide
SP3: Single-pot solid-phase-enhanced sample preparation
Ambic: Ammonium bicarbonate
TEAB: Triethyl ammonium bicarbonate
FA: Formic acid
ACN: Acetonitrile
UPLC: Ultra performance liquid chromatography
AGC: Automatic gain control
IT: Injection time
HCD: Higher-energy collision dissociation
NCE: Normalized collision energy
FRD: False discovery rate
PRIDE: Proteomics identification database
UNIPROT: Universal protein resource
GESTALT: Gene set analysis toolkit
cDNA: Complementary DNA
IDT: Integrated DNA technologies
PCR: Ploymerase chain reaction
PTM: Post translational modification
PSM: Peptide spectral match
SIS peptide: Stable isotope labeled synthetic peptide
DDCT: Delta-delta treshold cycle
gDNA: Genomic DNA
