# Supplementary material for: Quantitative proteome profiling reveals molecular hallmarks of egg quality in Atlantic halibut: impairments of transcription and protein folding impede protein and energy homeostasis during early development
Source: BMC Genomics. 2022 Sep 7;23:635. doi: 10.1186/s12864-022-08859-0 (PMC9450261; doi:10.1186/s12864-022-08859-0)
Supplement: Supplementary file 1 — Additional file 1: Fig. S1. Correlation of reproductive parameters with embryo survival and egg quality in halibut. Fig. S2. TMT labeling based LC-MS/MS quantification for candidate marker proteins. Fig. S3. TaqMan qPCR based relative quantification of gene expression for candidate marker proteins. Fig. S4. PRM based LC-MS/MS quantification of protein abundances for candidate marker proteins. Fig. S5. Results of counts and measurements of some mitochondrial parameters based on TEM imaging. Fig. S6. Some examples of mitochondrial observations in halibut eggs. Fig. S7. Biological samples and egg quality assessment procedure used in this study. Fig. S8. Experimental design. Fig. S9. Quantification of heavy peptides selected for use in PRM based LC-MS/MS. Table S1. List of females used in this study. Table S2. Differentially abundant proteins between good and poor quality halibut eggs based on peak area intensities obtained from the TMT based LC-MS/MS. Table S3. STRING protein-protein interaction networks enrichment analysis. Table S4. List of primers and probes used for relative quantification of gene expression and mtDNA quantification using TaqMan based qPCR. Table S5. List of reference peptides used for PRM based LC-MS/MS. [file 12864_2022_8859_MOESM1_ESM.zip › Supplementary Information.docx]

**SUPPLEMENTARY INFORMATION**

**Fig S1. Correlation of reproductive parameters with embryo survival and egg quality in halibut. Panel A.** Fertilization indicates the percentage of eggs showing visible embryonic cell division at 24 hpf. Normal cell division indicates the percentage of eggs bearing embryos with symmetrical cell division at 24 hpf. Buoyancy indicates egg density (g/cm^3^) measured in a buoyancy column at 1 hpf (see Methods). Fecundity indicates the volume of eggs in liters collected per batch. Survival indicates the percentage of eggs bearing embryos surviving to 12 dpf. **Panel B.** Quality indicates the quality of egg batches. Good quality (GQ) indicates that the percentage eggs bearing surviving embryos at 12 dpf was ≥ 72%; Poor quality (BQ) indicates that the percentage eggs bearing surviving embryos at 12 dpf was < 71%. **Panel C**. The range of survival observations for GQ and BQ eggs is shown (left side) and the Spearman's correlation coefficient (rho method) and corresponding significance values (*p* < 0.05) for the comparisons made in Panel A and B are given (right side). Summary values for each parameter representing egg batches used in this study are given in **Table S1**.

**Fig S2. TMT labeling based LC-MS/MS quantification for candidate marker proteins.** Each panel represents comparisons of protein abundances in peak area intensities between good (GQ) and poor quality (BQ) halibut eggs for 21 candidate marker proteins. All proteins differed significantly in abundance between good and poor quality eggs (Independent t-test *p* < 0.05, followed by Benjamini Hochberg correction for multiple tests *p* < 0.05). See **Table S2** for details of protein names.

**Fig S3. TaqMan qPCR based relative quantification of gene expression for candidate marker proteins.** Each panel represents comparisons of transcript abundances for 21 differentially abundant proteins between good (GQ) and poor quality (BQ) halibut eggs. TaqMan qPCR-2^-ΔΔCT^ mean relative quantification of gene expression was employed using halibut 18S ribosomal RNA (*18S*) as reference gene. Box plots indicate mean expression ratio (± SEM) with respect to the mean expression of the reference gene. Statistically significant differences are indicated with asterisks (Independent t-test *p* < 0.05 followed by Benjamini Hochberg correction for multiple tests *p* < 0.05).

**Fig S4. PRM based LC-MS/MS quantification of protein abundances for candidate marker proteins**. **Panel A.** Proteins down-regulated in poor quality (GQ) eggs. **Panel B.** Proteins down-regulate in poor quality (BQ) eggs. Each protein is represented by 1 to 3 reference peptides. The number of candidate marker proteins whom relative abundance has been validated with this method is limted to the avilibility of appropriate reference peptides. Details on selection of reference peptides are given in the **Material and Methods** section and **Fig S9**. Statistically significant differences are indicated with asterisks (Independent t-test *p* < 0.05 followed by Benjamini Hochberg correction for multiple tests *p* < 0.05).

**Fig S5. Results of counts and measurements of some mitochondrial parameters based on TEM imaging.** Each panel represents comparisons between good (GQ) and poor quality (BQ) halibut eggs. Statistically significant differences (*p* < 0.05) are indicated with asterisks and *p* values above each panel.

**Fig S6. Some examples of mitochondrial observations in halibut eggs.** Different panels represent different mitochondrial forms and movements that might be related to certain mitochondrial phenomenon observed in this study. **Panel A** shows well-formed examples of mitochondria with different size. **Panels B, C** and **D** show movements of two mitochondria towards fusion. **Panels E, F,** **G** and **H** exhibit examples of fused mitochondria. **Panels I, J, K** and **L** shows fused or not empty deformed vesicles containing double membranes which assumably used to be mitochondria. Similar mitochondrial events have been observed in both good and poor quality eggs groups. All images depicted in this figure were generated by the authors for use in this article. Scalebars indicate 0.5 µm at 50K magnification.

**Fig S7. Biological samples and egg quality assessment procedure used in this study.** A total of 10, 8 and 6 halibut egg batches were collected during the reproductive seasons of 2019, 2020, and 2021, respectively. Between the 3^rd^ and the 5^th^ batch of each season were targeted to ensure the fine tuning of spawning rhythm and stability of egg quality during the season in each female. Replicates of 0.5 ml eggs per spawn were snap frozen in liquid nitrogen at 1 hpf (at 1-cell stage) and stored at -80 ^o^C until analysis. Hundred milliliters of fertilized eggs from each spawn were incubated until hatching with daily care for mortality determination. Egg quality assessments were based on embryo survival prior hatching at 12 dpf. Egg batches with embryo survival rate of ≥ 72 were considered to be of good quality and those spawns with ≤ 71 embryo survival was considered to be of poor quality in all seasons (*See Material and Methods section for details*). The list of egg batches collected during each year and egg quality assessment parameters and classifications are given in **Table S1**. Except for the Atlantic halibut image which originated from the Havforskningsinstituttet media archive and not subject to copyright, all images depicted in this figure were generated by the authors for use in this article. hpf; hours post fertilization, dpf: days postfertilization

**Fig S8. Experimental design.** Each panel represents the flow of each experimental approach used in this study. The sequence of panels is set in the same order in which each experiment was carried out. Below each panel header the number of biological samples and the year in which they were collected are given. Except for the Atlantic halibut image which originated from the Havforskningsinstituttet media archive and not subject to copyright, all images depicted in this figure were generated by the authors for use in this article.

**Fig S9. Quantification of heavy peptides selected for use in PRM based LC-MS/MS.** Each panel contains scatter plots of abundances for each peptide representing differentially abundant proteins. Those results were obtained from the TMT based LC-MS/MS experiment. Blue markers represent values for good quality eggs while red markers indicate values for poor quality eggs. Significance of differences in abundance, the names and the amino acid sequences of each peptide are given above each panel. Clear clustering between good and poor quality eggs along with a *p* value of <0.05 (Independent t-test followed by Benjamini Hochberg correction for multiple testing *p* < 0.05), among other parameters were used for selection of these peptides where possible. See the **Material and Methods** section for details of other criteria used for reference peptide selection in this study.

**Table S1. List of females used in this study.** A list of females from which egg batches were obtained for this study are given. The sampling year, the group and female names, fecundity, fertilization rate, normal cell division rate, egg buoyancy, survival rate at 12 dpf, quality classification, and the application in which the samples were used are given in each column. * Egg batches with high fertilization and normal cell division rates but low survival rates.

| **Year** | **Group**  **(Batch)** | **Female**  **name** | **Fecundity (lt)** | **Fertilization**  **rate (%)** | **Normal**  **cell division %** | **Egg buoyancy**  **(Egg density g/cm3)** | **Survival**  **rate (%)** | **Quality** | **Application** |
| --- | --- | --- | --- | --- | --- | --- | --- | --- | --- |
| 2019 | Gr3* | Sophia | 1.7 | 93 | 89.96 |  | 44 | Poor | TMT, qPCR, PRM |
| 2019 | Gr11* | Sophia | 1.3 | 89 | 81.95 |  | 62 | Poor | TMT, qPCR |
| 2019 | Gr12 | Janette | 3.4 | 96 | 94.82 |  | 91 | Good | TMT, qPCR, PRM |
| 2019 | Gr15 | PinkFluid BBB0 | 0.9 | 54 | 47.62 |  | 49 | Poor | TMT, qPCR, PRM |
| 2019 | Gr17 | Green | 1.3 | 78 | 73.83 |  | 89 | Good | TMT, qPCR |
| 2019 | Gr19 | Pink2007 | 1.8 | 91 | 90.23 |  | 83 | Good | TMT, qPCR, PRM |
| 2019 | Gr20 | Black | 1.9 | 93 | 80.67 |  | 76 | Good | TMT, qPCR, PRM |
| 2019 | Gr21 | Green | 1.4 | 93 | 92.65 |  | 93 | Good | TMT, qPCR, PRM |
| 2019 | Gr22* | Red | 1.6 | 73 | 72.94 |  | 50 | Poor | TMT, qPCR, PRM |
| 2019 | Gr24 | Rangfrid | 1.25 | 56 | 24.14 |  | 57 | Poor | TMT, qPCR, PRM |
|  |  |  |  |  |  |  |  |  |  |
| 2020 | Gr2 | Janette | 2 | 94 | 91.18 | 1.0274 ± 0.00002 | 76 | Good | PRM, qPCR |
| 2020 | Gr4* | Sophia | 1 | 92 | 89.09 | 1.0277 ± 0.00001 | 36 | Poor | PRM, qPCR |
| 2020 | Gr8 | Pink2007 | 0.86 | 87 | 77.99 | 1.0278 ± 0.00002 | 79 | Good | PRM, qPCR |
| 2020 | Gr9* | Black | 0.7 | 78 | 66.67 | 1.0278 ± 0.00001 | 71 | Poor | PRM, qPCR |
| 2020 | Gr10 | Ny-liten | 0.5 | 43 | 42.79 | 1.0282 ± 0.00002 | 25 | Poor | PRM, qPCR |
| 2020 | Gr11* | Red | 0.75 | 79 | 73.08 | 1.0280 ± 0.00002 | 51 | Poor | PRM, qPCR |
| 2020 | Gr13 | Yellow | 1 | 79 | 77.91 | 1.0265 ± 0.00002 | 87 | Good | PRM, qPCR |
| 2020 | Gr14 | Green | 0.35 | 84 | 76.72 | 1.0275 ± 0.00001 | 85 | Good | PRM, qPCR |
|  |  |  |  |  |  |  |  |  |  |
| 2021 | Gr8 | Pink2007 | 1 | 85 | 80.79 |  | 72 | Good | TEM |
| 2021 | Gr9 | Black | 1.2 | 57 | 51.02 |  | 74 | Good | TEM |
| 2021 | Gr10 | Red | 1 | 66 | 61.29 |  | 55 | Poor | TEM |
| 2021 | Gr11* | Sissel | 0.6 | 87 | 86.70 |  | 43 | Poor | TEM |
| 2021 | Gr12* | Yellow | 1 | 72 | 70.95 |  | 53 | Poor | TEM |
| 2021 | Gr14 | Ozlem | 1.25 | 90 | 88.78 |  | 90 | Good | TEM |
|  |  |  |  |  |  |  |  |  |  |

**Table S2. Differentially abundant proteins between good and poor quality halibut eggs based on peak area intensities obtained from the TMT based LC-MS/MS.** List of the 115 proteins which were differentially regulated between good (GQ) and poor quality (BQ) eggs and whose distribution among various functional categories is illustrated in Fig 2. These include proteins up-regulated in poor quality eggs (BQ-upregulated, N = 51) and proteins down-regulated in poor quality eggs (BQ-downregulated, N = 64). For each protein, the NCBI gene IDs, NCBI accession numbers, associated protein names from human database, protein full names, functional categories (Fig 2), significance of differences in abundance (Independent t- test *p* < 0.05, followed by Benjamini Hochberg correction for multiple tests *p* < 0.05), relative abundance ratios (GQ/BQ and BQ/GQ, respectively), and regulation tendencies (BQ-upregulated or BQ-downregulated) are given in each column. Colour shading corresponds to that used to designate functional categories in **Fig 2**.

**Table S3. STRING protein-protein interaction networks enrichment analysis.** Network enrichment analyses for differentially regulated proteins (down-regulated in poor quality eggs, N = 64 and up-regulated in poor quality eggs) which were resolved in a STRING subnetworks. Gene Ontology (GO) Biological processes, molecular functions and cellular components, along with KEGG and Reactome pathways are given. Proteins were mapped against human database. GO-term IDs, description of processes and pathways, number of query proteins mapping to the total number of representative proteins present in the database and the false discovery rates (FDR) are given in each column. PPI enrichment *p* values indicating the existence of non-random nodes and therefore the significance of the observed edge numbers are given in the header panels.

| **Network Stats for proteins upregulated in good quality eggs N=64** | | | | |
| --- | --- | --- | --- | --- |
| Reference database: | | Human | | |
| number of nodes: | | 62 | | |
| number of edges: | | 65 | | |
| expected number of edges: | | 28 | | |
| average node degree: | | 2.1 | | |
| avg. local clustering coefficient: | | 0.316 | | |
| PPI enrichment p-value: | | 1.70E-09 | | |
|  | |  | | |
| **Biological Process (GO)** | | | | |
| ***GO-term*** | ***Description*** | | ***#Protein*** | ***FDR*** |
| [GO:1901575](http://amigo.geneontology.org/amigo/term/GO:1901575) | organic substance catabolic process | | 17 of 1609 | 1.90E-03 |
| [GO:1901566](http://amigo.geneontology.org/amigo/term/GO:1901566) | organonitrogen compound biosynthetic process | | 16 of 1370 | 1.90E-03 |
| [GO:1901564](http://amigo.geneontology.org/amigo/term/GO:1901564) | organonitrogen compound metabolic process | | 34 of 5281 | 1.90E-03 |
| [GO:0055114](http://amigo.geneontology.org/amigo/term/GO:0055114) | oxidation-reduction process | | 14 of 932 | 1.90E-03 |
| [GO:0055086](http://amigo.geneontology.org/amigo/term/GO:0055086) | nucleobase-containing small molecule metabolic process | | 11 of 662 | 1.90E-03 |
| [GO:0051186](http://amigo.geneontology.org/amigo/term/GO:0051186) | cofactor metabolic process | | 10 of 467 | 1.90E-03 |
| [GO:0044282](http://amigo.geneontology.org/amigo/term/GO:0044282) | small molecule catabolic process | | 9 of 388 | 1.90E-03 |
| [GO:0044281](http://amigo.geneontology.org/amigo/term/GO:0044281) | small molecule metabolic process | | 19 of 1779 | 1.90E-03 |
| [GO:0044248](http://amigo.geneontology.org/amigo/term/GO:0044248) | cellular catabolic process | | 18 of 1646 | 1.90E-03 |
| [GO:0043603](http://amigo.geneontology.org/amigo/term/GO:0043603) | cellular amide metabolic process | | 12 of 732 | 1.90E-03 |
| [GO:0034655](http://amigo.geneontology.org/amigo/term/GO:0034655) | nucleobase-containing compound catabolic process | | 9 of 394 | 1.90E-03 |
| [GO:0019752](http://amigo.geneontology.org/amigo/term/GO:0019752) | carboxylic acid metabolic process | | 13 of 854 | 1.90E-03 |
| [GO:0008152](http://amigo.geneontology.org/amigo/term/GO:0008152) | metabolic process | | 48 of 9569 | 1.90E-03 |
| [GO:0006732](http://amigo.geneontology.org/amigo/term/GO:0006732) | coenzyme metabolic process | | 8 of 297 | 1.90E-03 |
| [GO:0043604](http://amigo.geneontology.org/amigo/term/GO:0043604) | amide biosynthetic process | | 9 of 495 | 2.10E-03 |
| [GO:0017144](http://amigo.geneontology.org/amigo/term/GO:0017144) | drug metabolic process | | 10 of 622 | 2.10E-03 |
| [GO:1901135](http://amigo.geneontology.org/amigo/term/GO:1901135) | carbohydrate derivative metabolic process | | 13 of 1083 | 2.30E-03 |
| [GO:0044237](http://amigo.geneontology.org/amigo/term/GO:0044237) | cellular metabolic process | | 44 of 8797 | 2.30E-03 |
| [GO:0009066](http://amigo.geneontology.org/amigo/term/GO:0009066) | aspartate family amino acid metabolic process | | 4 of 55 | 2.40E-03 |
| [GO:0016071](http://amigo.geneontology.org/amigo/term/GO:0016071) | mRNA metabolic process | | 10 of 667 | 3.10E-03 |
| [GO:0034641](http://amigo.geneontology.org/amigo/term/GO:0034641) | cellular nitrogen compound metabolic process | | 31 of 5126 | 3.40E-03 |
| [GO:0006402](http://amigo.geneontology.org/amigo/term/GO:0006402) | mRNA catabolic process | | 6 of 207 | 3.40E-03 |
| [GO:0051188](http://amigo.geneontology.org/amigo/term/GO:0051188) | cofactor biosynthetic process | | 6 of 218 | 4.40E-03 |
| [GO:0009117](http://amigo.geneontology.org/amigo/term/GO:0009117) | nucleotide metabolic process | | 9 of 576 | 4.90E-03 |
| [GO:0046395](http://amigo.geneontology.org/amigo/term/GO:0046395) | carboxylic acid catabolic process | | 6 of 237 | 6.20E-03 |
| [GO:0034404](http://amigo.geneontology.org/amigo/term/GO:0034404) | nucleobase-containing small molecule biosynthetic process | | 5 of 149 | 6.20E-03 |
| [GO:0072521](http://amigo.geneontology.org/amigo/term/GO:0072521) | purine-containing compound metabolic process | | 8 of 478 | 6.50E-03 |
| [GO:0046434](http://amigo.geneontology.org/amigo/term/GO:0046434) | organophosphate catabolic process | | 5 of 152 | 6.50E-03 |
| [GO:0032787](http://amigo.geneontology.org/amigo/term/GO:0032787) | monocarboxylic acid metabolic process | | 8 of 477 | 6.50E-03 |
| [GO:0009987](http://amigo.geneontology.org/amigo/term/GO:0009987) | cellular process | | 58 of 14652 | 6.50E-03 |
| [GO:1901137](http://amigo.geneontology.org/amigo/term/GO:1901137) | carbohydrate derivative biosynthetic process | | 9 of 625 | 6.80E-03 |
| [GO:0006810](http://amigo.geneontology.org/amigo/term/GO:0006810) | transport | | 26 of 4130 | 7.40E-03 |
| [GO:0006913](http://amigo.geneontology.org/amigo/term/GO:0006913) | nucleocytoplasmic transport | | 6 of 263 | 8.30E-03 |
| [GO:0034248](http://amigo.geneontology.org/amigo/term/GO:0034248) | regulation of cellular amide metabolic process | | 7 of 379 | 8.40E-03 |
| [GO:0009108](http://amigo.geneontology.org/amigo/term/GO:0009108) | coenzyme biosynthetic process | | 5 of 167 | 8.40E-03 |
| [GO:0071704](http://amigo.geneontology.org/amigo/term/GO:0071704) | organic substance metabolic process | | 43 of 9135 | 9.90E-03 |
| [GO:0019637](http://amigo.geneontology.org/amigo/term/GO:0019637) | organophosphate metabolic process | | 11 of 1011 | 1.15E-02 |
| [GO:0009204](http://amigo.geneontology.org/amigo/term/GO:0009204) | deoxyribonucleoside triphosphate catabolic process | | 2 of 7 | 1.20E-02 |
| [GO:0009166](http://amigo.geneontology.org/amigo/term/GO:0009166) | nucleotide catabolic process | | 4 of 101 | 1.20E-02 |
| [GO:0000956](http://amigo.geneontology.org/amigo/term/GO:0000956) | nuclear-transcribed mRNA catabolic process | | 5 of 191 | 1.30E-02 |
| [GO:0072329](http://amigo.geneontology.org/amigo/term/GO:0072329) | monocarboxylic acid catabolic process | | 4 of 106 | 1.35E-02 |
| [GO:0044283](http://amigo.geneontology.org/amigo/term/GO:0044283) | small molecule biosynthetic process | | 8 of 569 | 1.41E-02 |
| [GO:0006807](http://amigo.geneontology.org/amigo/term/GO:0006807) | nitrogen compound metabolic process | | 40 of 8349 | 1.41E-02 |
| [GO:0006520](http://amigo.geneontology.org/amigo/term/GO:0006520) | cellular amino acid metabolic process | | 6 of 308 | 1.43E-02 |
| [GO:0072522](http://amigo.geneontology.org/amigo/term/GO:0072522) | purine-containing compound biosynthetic process | | 5 of 206 | 1.61E-02 |
| [GO:0050684](http://amigo.geneontology.org/amigo/term/GO:0050684) | regulation of mRNA processing | | 4 of 114 | 1.61E-02 |
| [GO:1901605](http://amigo.geneontology.org/amigo/term/GO:1901605) | alpha-amino acid metabolic process | | 5 of 209 | 1.68E-02 |
| [GO:0009058](http://amigo.geneontology.org/amigo/term/GO:0009058) | biosynthetic process | | 27 of 4714 | 1.77E-02 |
| [GO:0006554](http://amigo.geneontology.org/amigo/term/GO:0006554) | lysine catabolic process | | 2 of 10 | 1.80E-02 |
| [GO:0051170](http://amigo.geneontology.org/amigo/term/GO:0051170) | import into nucleus | | 4 of 122 | 1.89E-02 |
| [GO:0006733](http://amigo.geneontology.org/amigo/term/GO:0006733) | oxidoreduction coenzyme metabolic process | | 4 of 124 | 1.98E-02 |
| [GO:0006790](http://amigo.geneontology.org/amigo/term/GO:0006790) | sulfur compound metabolic process | | 6 of 343 | 2.15E-02 |
| [GO:0016575](http://amigo.geneontology.org/amigo/term/GO:0016575) | histone deacetylation | | 3 of 57 | 2.34E-02 |
| [GO:0009179](http://amigo.geneontology.org/amigo/term/GO:0009179) | purine ribonucleoside diphosphate metabolic process | | 3 of 58 | 2.42E-02 |
| [GO:1903311](http://amigo.geneontology.org/amigo/term/GO:1903311) | regulation of mRNA metabolic process | | 5 of 238 | 2.46E-02 |
| [GO:0046365](http://amigo.geneontology.org/amigo/term/GO:0046365) | monosaccharide catabolic process | | 3 of 59 | 2.46E-02 |
| [GO:0006518](http://amigo.geneontology.org/amigo/term/GO:0006518) | peptide metabolic process | | 7 of 497 | 2.46E-02 |
| [GO:0000288](http://amigo.geneontology.org/amigo/term/GO:0000288) | nuclear-transcribed mRNA catabolic process, deadenylation-dependent decay | | 3 of 59 | 2.46E-02 |
| [GO:0009141](http://amigo.geneontology.org/amigo/term/GO:0009141) | nucleoside triphosphate metabolic process | | 5 of 246 | 2.68E-02 |
| [GO:1901576](http://amigo.geneontology.org/amigo/term/GO:1901576) | organic substance biosynthetic process | | 26 of 4656 | 2.80E-02 |
| [GO:0008090](http://amigo.geneontology.org/amigo/term/GO:0008090) | retrograde axonal transport | | 2 of 16 | 3.24E-02 |
| [GO:0045055](http://amigo.geneontology.org/amigo/term/GO:0045055) | regulated exocytosis | | 8 of 691 | 3.33E-02 |
| [GO:0044238](http://amigo.geneontology.org/amigo/term/GO:0044238) | primary metabolic process | | 40 of 8808 | 3.35E-02 |
| [GO:0010629](http://amigo.geneontology.org/amigo/term/GO:0010629) | negative regulation of gene expression | | 13 of 1670 | 4.11E-02 |
| [GO:0009264](http://amigo.geneontology.org/amigo/term/GO:0009264) | deoxyribonucleotide catabolic process | | 2 of 19 | 4.11E-02 |
| [GO:0048024](http://amigo.geneontology.org/amigo/term/GO:0048024) | regulation of mRNA splicing, via spliceosome | | 3 of 77 | 4.20E-02 |
| [GO:0044242](http://amigo.geneontology.org/amigo/term/GO:0044242) | cellular lipid catabolic process | | 4 of 167 | 4.20E-02 |
| [GO:0046386](http://amigo.geneontology.org/amigo/term/GO:0046386) | deoxyribose phosphate catabolic process | | 2 of 20 | 4.31E-02 |
| [GO:0044249](http://amigo.geneontology.org/amigo/term/GO:0044249) | cellular biosynthetic process | | 25 of 4567 | 4.31E-02 |
| [GO:0006555](http://amigo.geneontology.org/amigo/term/GO:0006555) | methionine metabolic process | | 2 of 20 | 4.31E-02 |
| [GO:0090407](http://amigo.geneontology.org/amigo/term/GO:0090407) | organophosphate biosynthetic process | | 7 of 577 | 4.46E-02 |
| [GO:0009165](http://amigo.geneontology.org/amigo/term/GO:0009165) | nucleotide biosynthetic process | | 5 of 291 | 4.46E-02 |
| [GO:0009150](http://amigo.geneontology.org/amigo/term/GO:0009150) | purine ribonucleotide metabolic process | | 6 of 425 | 4.46E-02 |
| [GO:0009062](http://amigo.geneontology.org/amigo/term/GO:0009062) | fatty acid catabolic process | | 3 of 84 | 4.73E-02 |
|  |  | |  |  |
|  |  | |  |  |
|  |  | |  |  |
| **Molecular Function (GO)** | | | | |
| ***GO-term*** | ***Description*** | | ***#Protein*** | ***FDR*** |
| [GO:0016491](http://amigo.geneontology.org/amigo/term/GO:0016491) | oxidoreductase activity | | 12 of 716 | 7.70E-04 |
| [GO:0016853](http://amigo.geneontology.org/amigo/term/GO:0016853) | isomerase activity | | 6 of 147 | 1.40E-03 |
| [GO:0043022](http://amigo.geneontology.org/amigo/term/GO:0043022) | ribosome binding | | 4 of 50 | 2.70E-03 |
| [GO:0043021](http://amigo.geneontology.org/amigo/term/GO:0043021) | ribonucleoprotein complex binding | | 5 of 116 | 3.20E-03 |
| [GO:0003824](http://amigo.geneontology.org/amigo/term/GO:0003824) | catalytic activity | | 33 of 5592 | 3.20E-03 |
| [GO:0030619](http://amigo.geneontology.org/amigo/term/GO:0030619) | U1 snRNA binding | | 2 of 7 | 1.83E-02 |
| [GO:0047429](http://amigo.geneontology.org/amigo/term/GO:0047429) | nucleoside-triphosphate diphosphatase activity | | 2 of 10 | 2.87E-02 |
| [GO:0001846](http://amigo.geneontology.org/amigo/term/GO:0001846) | opsonin binding | | 2 of 14 | 4.52E-02 |
|  |  | |  |  |
|  |  | |  |  |
| **Cellular Component (GO)** | | | | |
| ***GO-term*** | ***Description*** | | ***#Protein*** | ***FDR*** |
| [GO:0005737](http://amigo.geneontology.org/amigo/term/GO:0005737) | cytoplasm | | 60 of 11238 | 4.26E-10 |
| [GO:0005739](http://amigo.geneontology.org/amigo/term/GO:0005739) | mitochondrion | | 20 of 1531 | 4.58E-06 |
| [GO:0043231](http://amigo.geneontology.org/amigo/term/GO:0043231) | intracellular membrane-bounded organelle | | 53 of 10365 | 7.24E-06 |
| [GO:0005622](http://amigo.geneontology.org/amigo/term/GO:0005622) | intracellular | | 61 of 14286 | 7.24E-06 |
| [GO:0043229](http://amigo.geneontology.org/amigo/term/GO:0043229) | intracellular organelle | | 57 of 12193 | 7.30E-06 |
| [GO:0070013](http://amigo.geneontology.org/amigo/term/GO:0070013) | intracellular organelle lumen | | 36 of 5162 | 7.86E-06 |
| [GO:0043227](http://amigo.geneontology.org/amigo/term/GO:0043227) | membrane-bounded organelle | | 54 of 11244 | 1.49E-05 |
| [GO:0005759](http://amigo.geneontology.org/amigo/term/GO:0005759) | mitochondrial matrix | | 9 of 463 | 4.70E-04 |
| [GO:0005623](http://amigo.geneontology.org/amigo/term/GO:0005623) | cell | | 61 of 16271 | 3.90E-03 |
| [GO:0005829](http://amigo.geneontology.org/amigo/term/GO:0005829) | cytosol | | 29 of 4958 | 5.30E-03 |
| [GO:1904724](http://amigo.geneontology.org/amigo/term/GO:1904724) | tertiary granule lumen | | 3 of 55 | 1.84E-02 |
| [GO:0120111](http://amigo.geneontology.org/amigo/term/GO:0120111) | neuron projection cytoplasm | | 3 of 71 | 3.47E-02 |
| [GO:0099568](http://amigo.geneontology.org/amigo/term/GO:0099568) | cytoplasmic region | | 6 of 402 | 3.51E-02 |
| [GO:0005740](http://amigo.geneontology.org/amigo/term/GO:0005740) | mitochondrial envelope | | 8 of 722 | 3.73E-02 |
| [GO:0120114](http://amigo.geneontology.org/amigo/term/GO:0120114) | Sm-like protein family complex | | 3 of 78 | 3.75E-02 |
| [GO:0000932](http://amigo.geneontology.org/amigo/term/GO:0000932) | P-body | | 3 of 81 | 3.94E-02 |
|  |  | |  |  |
|  |  | |  |  |
|  |  | |  |  |
|  |  | |  |  |
| **KEGG Pathways** | | | | |
| ***Pathway*** | ***Description*** | | ***#Protein*** | ***FDR*** |
| [hsa01100](https://www.kegg.jp/kegg-bin/show_pathway?hsa01100) | Metabolic pathways | | 19 of 1250 | 6.13E-07 |
| [hsa03018](https://www.kegg.jp/kegg-bin/show_pathway?hsa03018) | RNA degradation | | 5 of 77 | 2.80E-04 |
| [hsa00280](https://www.kegg.jp/kegg-bin/show_pathway?hsa00280) | Valine, leucine and isoleucine degradation | | 3 of 48 | 1.38E-02 |
| [hsa00071](https://www.kegg.jp/kegg-bin/show_pathway?hsa00071) | Fatty acid degradation | | 3 of 44 | 1.38E-02 |
| [hsa04217](https://www.kegg.jp/kegg-bin/show_pathway?hsa04217) | Necroptosis | | 4 of 155 | 2.77E-02 |
| [hsa00010](https://www.kegg.jp/kegg-bin/show_pathway?hsa00010) | Glycolysis / Gluconeogenesis | | 3 of 68 | 2.77E-02 |
|  |  | |  |  |
|  |  | |  |  |
|  |  | |  |  |
| **Reactome Pathways** | | | | |
| ***Pathway*** | ***Description*** | | ***#Protein*** | ***FDR*** |
| [HSA-1430728](https://reactome.org/content/detail/R-HSA-1430728) | Metabolism | | 19 of 2032 | 3.30E-03 |
|  |  | |  |  |

| **Network Stats for proteins upregulated in poor quality eggs N=51 Human DB** | | | |
| --- | --- | --- | --- |
| Reference database: | Human |  |  |
| Number of nodes: | 50 | | |
| Number of edges: | 36 | | |
| Expected number of edges: | 20 | | |
| Average node degree: | 1.44 | | |
| Avg. local clustering coefficient: | 0.405 | | |
| PPI enrichment p-value: | 5.74E-04 | | |
|  |  | | |
| **Biological Process (GO)** | | | |
| ***GO-term*** | ***Description*** | ***#Protein*** | ***FDR*** |
| [GO:0071704](http://amigo.geneontology.org/amigo/term/GO:0071704) | organic substance metabolic process | 39 of 9135 | 9.00E-03 |
| [GO:0044238](http://amigo.geneontology.org/amigo/term/GO:0044238) | primary metabolic process | 38 of 8808 | 9.00E-03 |
| [GO:0044237](http://amigo.geneontology.org/amigo/term/GO:0044237) | cellular metabolic process | 38 of 8797 | 9.00E-03 |
| [GO:0009987](http://amigo.geneontology.org/amigo/term/GO:0009987) | cellular process | 49 of 14652 | 9.00E-03 |
| [GO:0008152](http://amigo.geneontology.org/amigo/term/GO:0008152) | metabolic process | 40 of 9569 | 9.00E-03 |
| [GO:0044403](http://amigo.geneontology.org/amigo/term/GO:0044403) | symbiont process | 9 of 650 | 9.10E-03 |
| [GO:0008380](http://amigo.geneontology.org/amigo/term/GO:0008380) | RNA splicing | 7 of 391 | 1.31E-02 |
| [GO:0006091](http://amigo.geneontology.org/amigo/term/GO:0006091) | generation of precursor metabolites and energy | 7 of 400 | 1.32E-02 |
| [GO:0016032](http://amigo.geneontology.org/amigo/term/GO:0016032) | viral process | 8 of 571 | 1.44E-02 |
| [GO:0007005](http://amigo.geneontology.org/amigo/term/GO:0007005) | mitochondrion organization | 7 of 424 | 1.44E-02 |
| [GO:0006839](http://amigo.geneontology.org/amigo/term/GO:0006839) | mitochondrial transport | 5 of 223 | 3.40E-02 |
|  |  |  |  |
|  |  |  |  |
| **Cellular Component (GO)** | | | |
| ***GO-term*** | ***Description*** | ***#Protein*** | ***FDR*** |
| [GO:0005750](http://amigo.geneontology.org/amigo/term/GO:0005750) | mitochondrial respiratory chain complex III | 3 of 13 | 2.00E-03 |
| [GO:0005743](http://amigo.geneontology.org/amigo/term/GO:0005743) | mitochondrial inner membrane | 8 of 456 | 2.00E-03 |
| [GO:0043231](http://amigo.geneontology.org/amigo/term/GO:0043231) | intracellular membrane-bounded organelle | 40 of 10365 | 2.70E-03 |
| [GO:0031966](http://amigo.geneontology.org/amigo/term/GO:0031966) | mitochondrial membrane | 9 of 679 | 2.70E-03 |
| [GO:0005737](http://amigo.geneontology.org/amigo/term/GO:0005737) | cytoplasm | 42 of 11238 | 2.70E-03 |
| [GO:0031967](http://amigo.geneontology.org/amigo/term/GO:0031967) | organelle envelope | 11 of 1146 | 2.90E-03 |
| [GO:0005829](http://amigo.geneontology.org/amigo/term/GO:0005829) | cytosol | 25 of 4958 | 3.00E-03 |
| [GO:1904813](http://amigo.geneontology.org/amigo/term/GO:1904813) | ficolin-1-rich granule lumen | 4 of 125 | 5.70E-03 |
| [GO:0098800](http://amigo.geneontology.org/amigo/term/GO:0098800) | inner mitochondrial membrane protein complex | 4 of 128 | 5.80E-03 |
| [GO:0005739](http://amigo.geneontology.org/amigo/term/GO:0005739) | mitochondrion | 12 of 1531 | 6.00E-03 |
| [GO:0070013](http://amigo.geneontology.org/amigo/term/GO:0070013) | intracellular organelle lumen | 24 of 5162 | 1.00E-02 |
| [GO:0043229](http://amigo.geneontology.org/amigo/term/GO:0043229) | intracellular organelle | 42 of 12193 | 1.00E-02 |
| [GO:0032991](http://amigo.geneontology.org/amigo/term/GO:0032991) | protein-containing complex | 23 of 4792 | 1.00E-02 |
| [GO:0005622](http://amigo.geneontology.org/amigo/term/GO:0005622) | intracellular | 46 of 14286 | 1.00E-02 |
| [GO:0071013](http://amigo.geneontology.org/amigo/term/GO:0071013) | catalytic step 2 spliceosome | 3 of 99 | 1.83E-02 |
| [GO:1902494](http://amigo.geneontology.org/amigo/term/GO:1902494) | catalytic complex | 9 of 1295 | 3.75E-02 |
| [GO:0071011](http://amigo.geneontology.org/amigo/term/GO:0071011) | precatalytic spliceosome | 2 of 41 | 3.75E-02 |
| [GO:0005634](http://amigo.geneontology.org/amigo/term/GO:0005634) | nucleus | 27 of 6892 | 3.75E-02 |
|  |  |  |  |
|  |  |  |  |
|  |  |  |  |
| **Local STRING network cluster** | | | |
| ***Cluster*** | ***Description*** | ***#Protein*** | ***FDR*** |
| [CL:15986](https://string-db.org/cgi/network.pl?network_cluster_id=CL:15986&input_query_species=9606) | mRNA Splicing | 7 of 185 | 1.30E-04 |
| [CL:22476](https://string-db.org/cgi/network.pl?network_cluster_id=CL:22476&input_query_species=9606) | mitochondrial respiratory chain complex III | 3 of 6 | 1.60E-04 |
| [CL:22328](https://string-db.org/cgi/network.pl?network_cluster_id=CL:22328&input_query_species=9606) | respirasome | 5 of 94 | 2.50E-04 |
| [CL:22327](https://string-db.org/cgi/network.pl?network_cluster_id=CL:22327&input_query_species=9606) | Oxidative phosphorylation | 6 of 153 | 2.50E-04 |
| [CL:15989](https://string-db.org/cgi/network.pl?network_cluster_id=CL:15989&input_query_species=9606) | mRNA Splicing - Major Pathway | 6 of 157 | 2.50E-04 |
| [CL:15992](https://string-db.org/cgi/network.pl?network_cluster_id=CL:15992&input_query_species=9606) | mRNA Splicing - Major Pathway | 5 of 146 | 1.20E-03 |
| [CL:22417](https://string-db.org/cgi/network.pl?network_cluster_id=CL:22417&input_query_species=9606) | Complex I biogenesis | 2 of 11 | 9.00E-03 |
| [CL:15994](https://string-db.org/cgi/network.pl?network_cluster_id=CL:15994&input_query_species=9606) | mRNA Splicing - Major Pathway | 4 of 140 | 9.00E-03 |
| [CL:13515](https://string-db.org/cgi/network.pl?network_cluster_id=CL:13515&input_query_species=9606) | protein neddylation | 2 of 11 | 9.00E-03 |
|  |  |  |  |
|  |  |  |  |
| **KEGG Pathways** | | | |
| ***Pathway*** | ***Description*** | ***#Protein*** | ***FDR*** |
| [hsa05010](https://www.kegg.jp/kegg-bin/show_pathway?hsa05010) | Alzheimer's disease | 5 of 168 | 4.10E-03 |
| [hsa05012](https://www.kegg.jp/kegg-bin/show_pathway?hsa05012) | Parkinson's disease | 4 of 142 | 7.20E-03 |
| [hsa04714](https://www.kegg.jp/kegg-bin/show_pathway?hsa04714) | Thermogenesis | 5 of 228 | 7.20E-03 |
| [hsa01100](https://www.kegg.jp/kegg-bin/show_pathway?hsa01100) | Metabolic pathways | 11 of 1250 | 7.20E-03 |
| [hsa00190](https://www.kegg.jp/kegg-bin/show_pathway?hsa00190) | Oxidative phosphorylation | 4 of 131 | 7.20E-03 |
| [hsa04260](https://www.kegg.jp/kegg-bin/show_pathway?hsa04260) | Cardiac muscle contraction | 3 of 76 | 9.50E-03 |
| [hsa05016](https://www.kegg.jp/kegg-bin/show_pathway?hsa05016) | Huntington's disease | 4 of 193 | 1.20E-02 |
| [hsa00020](https://www.kegg.jp/kegg-bin/show_pathway?hsa00020) | Citrate cycle (TCA cycle) | 2 of 30 | 1.99E-02 |
| [hsa03040](https://www.kegg.jp/kegg-bin/show_pathway?hsa03040) | Spliceosome | 3 of 130 | 2.79E-02 |
| [hsa04932](https://www.kegg.jp/kegg-bin/show_pathway?hsa04932) | Non-alcoholic fatty liver disease (NAFLD) | 3 of 149 | 3.63E-02 |
|  |  |  |  |
|  |  |  |  |
|  |  |  |  |
| **Reactome Pathways** | | | |
| ***Pathway*** | ***Description*** | ***#Protein*** | ***FDR*** |
| [HSA-1428517](https://reactome.org/content/detail/R-HSA-1428517) | The citric acid (TCA) cycle and respiratory electron transport | 7 of 173 | 1.10E-04 |
| [HSA-163200](https://reactome.org/content/detail/R-HSA-163200) | Respiratory electron transport, ATP synthesis by chemiosmotic coupling, and heat production by uncoupling proteins. | 6 of 123 | 1.40E-04 |
| [HSA-611105](https://reactome.org/content/detail/R-HSA-611105) | Respiratory electron transport | 5 of 100 | 7.10E-04 |
| [HSA-72203](https://reactome.org/content/detail/R-HSA-72203) | Processing of Capped Intron-Containing Pre-mRNA | 6 of 234 | 2.40E-03 |
| [HSA-72163](https://reactome.org/content/detail/R-HSA-72163) | mRNA Splicing - Major Pathway | 5 of 178 | 6.10E-03 |
| [HSA-1430728](https://reactome.org/content/detail/R-HSA-1430728) | Metabolism | 14 of 2032 | 1.87E-02 |
| [HSA-1169408](https://reactome.org/content/detail/R-HSA-1169408) | ISG15 antiviral mechanism | 3 of 69 | 3.11E-02 |
| [HSA-8953854](https://reactome.org/content/detail/R-HSA-8953854) | Metabolism of RNA | 7 of 652 | 4.03E-02 |
|  |  |  |  |
|  |  |  |  |
|  |  |  |  |
|  |  |  |  |
|  |  |  |  |
|  |  |  |  |
| **INTERPRO Protein Domains and Features** | | | |
| ***Domain*** | ***Description*** | ***#Protein*** | ***FDR*** |
| [IPR016024](https://www.ebi.ac.uk/interpro/entry/IPR016024) | Armadillo-type fold | 7 of 333 | 4.50E-03 |
| [IPR011989](https://www.ebi.ac.uk/interpro/entry/IPR011989) | Armadillo-like helical | 5 of 183 | 1.10E-02 |

**Table S4.** **List of primers and probes used for relative quantification of gene expression and mtDNA quantification using TaqMan based qPCR.** Names, sequences, length, GC content and annealing temperatures of primers and probes along with expected amplicon size in base pairs (bp) are given in each column.

| **Name** | **Sequence** | **Length** | **GC** | **Tm** | **Amplicon (bp)** |
| --- | --- | --- | --- | --- | --- |
| *18S*_Forward | TTGGCGAGGACCTGACTGTATTTCCCG | 27 | 55.6 | 62.1 | 146 |
| *18S*_Reverse | ACCAAGAGGGCAGGAGAACTCACTGAGG | 28 | 57.1 | 62.8 |  |
| *18S*_Probe | TGCATGATGGTCACCACCCGCTCCACCT | 28 | 60.7 | 66.8 |  |
| *mt-nd5*_Forward | GGCTTGAATAGCAGCAAACCTAAACTCCTGG | 31 | 48.4 | 61.4 | 154 |
| *mt-nd5*_Reverse | AGCGGAGGGTAGTCAAGGGTGAAGTCC | 27 | 59.3 | 62.9 |  |
| *mt-nd5*_Probe | ACTCATCATTGCAGCAACTGGCAAATCGGCTC | 32 | 50 | 64.8 |  |
| *cap1*_Forward | TTGTAAGAAGTTGGGGCTGGTGTTTGACC | 29 | 48.3 | 61.1 | 144 |
| *cap1*_Reverse | TGGCTCAGGTAAATGTGGCAACCGTC | 26 | 53.8 | 61.4 |  |
| *cap1*_Probe | CGTCGTCGGCATCGTGGAGATCATCAACTGC | 31 | 58.1 | 65.9 |  |
| *dhrs9*_Forward | AGGGAGAAGACGATCTGAAGAAGTCCTGC | 29 | 51.7 | 61.1 | 139 |
| *dhrs9*_Reverse | ACTACAGCCCATAAACCTCGCTCGCC | 26 | 57.7 | 62.4 |  |
| *dhrs9*_Probe | CCAGCAGCCTGACCACCATGCACCTCGAT | 29 | 62.1 | 67.2 |  |
| *gcn1*_Forward | TGTGCTGAAGTAACACACTCCGCAGCTC | 28 | 53.6 | 62.8 | 150 |
| *gcn1*_Reverse | ACCCAACCATCCGCAAGAACATTACCACC | 29 | 51.7 | 62.7 |  |
| *gcn1*_Probe | AGTTCACCAACACAGCCCGCTGACGCC | 27 | 63 | 66.8 |  |
| *ghitm*_Forward | ATGTGTGCCCCAAGCGAGAAGTTCC | 25 | 56 | 61.4 | 137 |
| *ghitm*_Reverse | GCCACTGAGTAAAGACCTGCTCCAAATGC | 29 | 51.7 | 61.7 |  |
| *ghitm*_Probe | TCGGATCAATGTTCCTGCCGCCCAGCTC | 28 | 60.7 | 65.9 |  |
| *uqcrfs1*_Forward | ACATCAGAGCGACGATAGTCTCCAAAGTCAG | 31 | 48.4 | 61.3 | 142 |
| *uqcrfs1*_Reverse | TGCAGGCAGCGAAACACTCCGTTAAG | 26 | 53.8 | 61.8 |  |
| *uqcrfs1*_Probe | AGCAGCAGCGGGCACCAGCTCCTT | 24 | 66.7 | 66.8 |  |
| *gatd3a*_Forward | GCTCAGCAGGCAACCACCTCTTCTTC | 26 | 57.7 | 61.6 | 138 |
| *gatd3a*_Reverse | CGCATTCATGTGCAACGCTCCCATC | 25 | 56 | 61.4 |  |
| *gatd3a*_Probe | TCAAGCCAGCTTCAGCACGTCCCGCA | 26 | 61.5 | 66.4 |  |
| *fbp1a*_Forward | TGCTTCGATCCTTTGGACGGCTCCTC | 26 | 57.7 | 62.4 | 101 |
| *fbp1a*_Reverse | TCACACGGCTCATCGTCTGTGGTCTTTC | 28 | 53.6 | 62.4 |  |
| *fbp1a*_Probe | TCGACTGTCTCGTCTCCATCGGCACCA | 27 | 59.3 | 64.5 |  |
| *atp5f1b*_Forward | GACCCACCATGTAGAACGCTTGCTCC | 26 | 57.7 | 61.5 | 148 |
| *atp5f1b*_Reverse | TTCTTGTCGCAGCCTTTCCAGGTCG | 25 | 56 | 61.4 |  |
| *atp5f1b*_Probe | TGTTTCCTTGAGAGGCACCAGCTTGCCCAT | 30 | 53.3 | 65.3 |  |
| *acylb*_Forward | TGTCGTCCACTGCAACCATCAGCCTC | 26 | 57.7 | 62.8 | 153 |
| *acylb*_Reverse | AGCCGTTTGTTCCACACACACAGGAAG | 27 | 51.9 | 61.6 |  |
| *acylb*_Probe | CGTCCACGTCGCCCACCTCCACTCCT | 26 | 69.2 | 67.2 |  |
| *gcdh*_Forward | GAGCTGATCTGGCTTCACCACGAGAC | 26 | 57.7 | 61 | 117 |
| *gcdh*_Reverse | GCCGTGCAGAATCGCTTTTCCTATGC | 26 | 53.8 | 61.1 |  |
| *gcdh*_Probe | TGCTCCTGCGTGAGGCTGTCCCAGTCA | 27 | 63 | 66.7 |  |
| *mt-atp6*_Forward | GCCCTCATCCCTGTACTTATT | 21 | 47.6 | 57.9 | 94 |
| *mt-atp6*_Reverse | CTGCTGTGAGATTTGCTGTTAG | 22 | 45.5 | 58.4 |  |
| *mt-atp6*_Probe | TCATTCGACCCTTAGCTCTCGGTGT | 25 | 64.6 | 52 |  |
| *uqcrb*_Forward | TTAACATGAAGCAGCAGATCC | 21 | 42.9 | 50.5 | 112 |
| *uqcrb*_Reverse | TCTATTTCTTTACGCTCACGG | 21 | 42.9 | 50.1 |  |
| *uqcrb*_Probe | AGGATATCAGCTACCTGTCACCATATCTGAA | 31 | 41.9 | 58.3 |  |
| *cyc1*_Forward | AACATCTCAGTAAGTCGTCTGAAGACCC | 28 | 46.4 | 58 | 133 |
| *cyc1*_Reverse | CAACCTTGTGCTTTCCACCATTCTCC | 26 | 50 | 58.5 |  |
| *cyc1*_Probe | CATTTGTCCAGAAGTGTGCCCAGTGCC | 27 | 55.6 | 62.2 |  |
| *fh*_Forward | AGCATTTCACTTGGCTTCACCCAC | 24 | 50 | 58.1 | 105 |
| *fh*_Reverse | CAAGACGGCTCACAAGAAAGGCAC | 24 | 54.2 | 58.7 |  |
| *fh*_Probe | TGCTCCTCGGTCACGTATCCCAGCTCAA | 28 | 57.1 | 64.4 |  |
| *pdia4*_Forward | CGTCCAAACCCCAACACTTTTCCTC | 25 | 52 | 58.4 | 100 |
| *pdia4*_Reverse | TCCATCCCTCAGTACCCTCTGTACTC | 26 | 53.8 | 58.1 |  |
| *pdia4*_Probe | TCCAGGTAGTAATTGATCTGCACGGCAGCCA | 31 | 51.6 | 64.4 |  |
| *ppid*_Forward | CGCAGTTCTTCATCACCACTGTCC | 24 | 54.2 | 58 | 101 |
| *ppid*_Reverse | CTCCAGCATTTTTACGACACCCATTCC | 27 | 48.1 | 58.5 |  |
| *ppid*_Probe | CCCTCACTTAGATGATAAGCACGTTGTCTTCGG | 33 | 48.5 | 62 |  |
| *phb*_Forward | GACGGGAACGGCAATCAAAGATGATAG | 27 | 48.1 | 58.2 | 102 |
| *phb*_Reverse | TCGACAGGTTCAGAGGAGTGCAAG | 24 | 54.2 | 58.4 |  |
| *phb*_Probe | TGATGAAGTGAGTCCCTTCACCAGCAACAGT | 31 | 48.4 | 62.9 |  |
| *vdac*_Forward | CTGTACCTCCCACCTACATTGACCTTG | 27 | 51.9 | 58.4 | 116 |
| *vdac*_Reverse | CTCCAGTCCATTGTCCGACTTTGTTTTC | 28 | 46.4 | 58.5 |  |
| *vdac*_Probe | TGGCTTCGGGCTCATCAAGCTGGACC | 26 | 61.5 | 64.5 |  |
| *mecr*_Forward | CAGGATTTTAACCAGGACATCCTTTGCAC | 29 | 44.8 | 58.6 | 107 |
| *mecr*_Reverse | GCTCAGCCCTTCAGTACAGACAACAC | 26 | 53.8 | 59.5 |  |
| *mecr*_Probe | TGGGTGGCAAATCTATATCTTCCAACTGGACGAC | 34 | 47.1 | 62.8 |  |
| *gfm1*_Forward | TCTCACCTGTCCAACAATCTCTGTTTCC | 28 | 46.4 | 58.6 | 106 |
| *gfm1*_Reverse | TAATGTGACGATCAGACCTGAAGCTGCC | 28 | 50 | 60.5 |  |
| *gfm1*_Probe | TGAGCCAACACGTCGTCCGCTGAG | 24 | 62.5 | 63.2 |  |
| *sod1*_Forward | ACACAAATGGGTGCATGAGTGCAG | 24 | 50 | 58.4 | 107 |
| *sod1*_Reverse | AGTCACATTCCCCAGGTCTCCAAC | 24 | 54.2 | 58.1 |  |
| *sod1*_Probe | TGCTGGTCCTACTGATGCAGACAGGCAC | 28 | 57.1 | 63.7 |  |
|  |  |  |  |  |  |

**Table S5.** **List of reference peptides used for PRM based LC-MS/MS.** Names, amino acid sequences, length in amino acids (aa), charge, peptide spectral matches (PSMs), miscleavage potentials, significant differences (*p* < 0.05) in peptide abundance between good and poor quality eggs (based on TMT labeling based LC-MS/MS), suitability for PRM and hydrophobicity rates (%) (based on Peptide Synthesis and Proteotypic Peptide Analysis, ThermoFisher Scientific) are given in each column. See the **Material and Methods** section for details on selection of reference peptides used in this study.

| **Protein** | **Peptide** | **Length (aa)** | **Charge** | **PSMs** | **Miscleavage** | ***p* < 0.05** | **Suitablity for PRM** | **Hydrophobicty (%)** |
| --- | --- | --- | --- | --- | --- | --- | --- | --- |
| DHRS9_5 | NVETLWA(K) | 8 | 2 | 2 | 0 | 0.043 | YES | 25.49 |
| DHRS9_6 | VVNVASVFG( R) | 10 | 2 | 2 | 0 | 0.027 | YES | 25.11 |
| DHRS9_7 | YSPGWDA(K) | 8 | 2 | 2 | 0 | 0.002 | YES | 18.96 |
| GATD3A_1 | DDKYPDTTGTAEAINQLGC(K) | 20 | 3 | 2 | 0 | 0.005 | YES | 25.18 |
| GATD3A_2 | GDIQDLS(K) | 8 | 2 | 2 | 0 | 0.007 | YES | 14.9 |
| GATD3A_3 | NVLVESA(R) | 8 | 2 | 2 | 0 | - | YES | 16.73 |
| MT-ND5_1 | AIEAANRPLVSSTSNIQ( R) | 18 | 3 | 2 | 0 | 0.103 | YES | 27.25 |
| MT-ND5_2 | FSPLSPINENNPAVINPLK( R) | 20 | 3 or 4 | 4 | 1 | - | YES | 36.65 |
| MT-ND5_3 | FSPLSPINENNPAVINPL(K) | 19 | 3 or 4 | 4 | 0 | - | YES | 38.5 |
| CAP1_7 | NALFNSIC(K) | 9 | 2 or 3 | 2 | 0 | 0.046 | YES | 26.38 |
| CAP1_8 | TGPVASAGAAPP(R) | 13 | 2 | 4 | 0 | 0.096 | YES | 13.44 |
| GCN1_3 | LTSPEALRPSVVNITGPLI(R) | 20 | 3 | 2 | 0 | 0.049 | YES | 39.42 |
| GCN1_4 | FVIQGAGS(K) | 9 | 2 | 2 | 0 | - | YES | 18.47 |
| GCN1_5 | IIIDDLLEAT( R) | 11 | 2 | 2 | 0 | 0.045 | YES | 39.06 |
| FBP1_1 | GTGELTTLLNAVGTAV(K) | 17 | 3 | 4 | 0 | 0.031 | YES | 39.25 |
| FBP1_2 | SSFTSCVLVSEEND(K) | 15 | 3 | 2 | 0 | 0.494 | YES | 27.6 |
| UQCRFS1_2 | LTDIPEG(K) | 8 | 2 | 2 | 0 | - | YES | 15.11 |
| UQCRFS1_2 | TVVSQFISSMSASADVLALS(K) | 21 | 3 or 4 | 2 | 0 | 0.037 | YES | 45.21 |
| UQCRFS1_3 | EIATEEAVNLAELRDPQHDKD(R) | 22 | 4 | - | 0 | - | YES | 32.46 |
| GHITM_1 | AETHPLYGVQ(K) | 11 | 3 | 2 | 0 | - | YES | 18.79 |
| GHITM_2 | EAAFEPATDTAI( R) | 13 | 2 or 3 | 4 | 0 | 0.049 | YES | 25.13 |
| GHITM_3 | SVIWPQYV(K) | 9 | 2 | 5 | 0 | - | YES | 32.65 |
